# Ferrielectric-mediated morphotropic phase boundaries in Bi-based polar perovskites

**DOI:** 10.1038/s41598-019-40724-1

**Published:** 2019-03-11

**Authors:** Yuuki Kitanaka, Masaru Miyayama, Yuji Noguchi

**Affiliations:** School of Engineering, The University of Tokyo, 7-3-1 Hongo, Bunkyo-ku, Tokyo, 113-856 Japan

## Abstract

Spontaneous polarization (*P*_s_) in ferroelectrics has provided the impetus to develop piezoelectric devices such as sensors, actuators and diagnostic imaging transducers. Widely used lead-based perovskites exhibit a composition-driven phase diagram involving a transition region, known as a morphotropic phase boundary, where the ferroelectric structure changes dramatically and the piezoelectric activity is maximal. In some perovskites, ferroic polarization coexists with nonpolar rotations of octahedra, suggesting an unprecedented phase diagram. Here, we show morphotropic phase boundaries, where ‘ferrielectric’ appears as a bridging phase between ferroelectrics with rhombohedral and tetragonal symmetries in Bi_1/2_Na_1/2_TiO_3_-based perovskites. Neutron diffraction analysis demonstrates that the intermediate ferrielectric displays a small *P*_s_ resulting from up and down polarizations coupled with an in-phase TiO_6_ rotation. Our *ab initio* calculations indicate that a staggered Bi-O conformation at an appropriate chemical pressure delivers the ferrielectric-mediated phase boundaries, which provides a promising platform for (multi)ferroic materials with enhanced physical properties.

## Introduction

Because functions of ferroelectrics are governed by spontaneous polarization (*P*_s_) in response to external stimuli, the majority of studies have focused on controlling polar lattice distortions^1–3^. In lead-based perovskites, e.g., Pb(Zr, Ti)O_3_, a composition-driven transition region, known as a morphotropic phase boundary (MPB), separates tetragonal and rhombohedral ferroelectrics^4,5^, between which an intermediated monoclinic appears as a bridging phase^6–9^. In the vicinity of the MPBs, a piezoelectric response is markedly enhanced because of the symmetry-allowed polarization rotation^10–13^. The nature of these phase boundaries where ferroelectric instabilities compete with each other can be explained by the intrinsic high-pressure MPBs in PbTiO_3_ tuned through a chemical pressure^9^.

For perovskite oxides and related materials with two-dimensional interfaces, a polar atomic configuration is triggered by the condensation of rotation instabilities of oxygen octahedra^14–18^. In a wide range of simple perovskites, octahedral rotations are ubiquitous; however, a system with multiple instabilities is relatively rare^19,20^. In some perovskites, such as the Bi_1/2_Na_1/2_TiO_3_–BaTiO_3_ system^21^, ferroic polarization coexists with nonpolar modes of TiO_6_ octahedra, suggesting a distinct phase diagram where ferroelectrics with rhombohedral *R*3*c* (or monoclinic *Cc*) and tetragonal *P*4*mm* are mediated by a ferroic phase involving TiO_6_ rotations^22–25^. Despite several decades of intensive research, it remains challenging to identify clear, unambiguous MPBs in which the subtle but important distortions participate because of a structural complexity partially owing to compositional inhomogeneity and/or of a detection limit of analytical methods^21,23–30^, see Supplementary Table 1. Moreover, there has been no research on the electronic origin of MPBs where abrupt changes in crystal structure, symmetry and atomic reconstruction are derived from orbital interactions.

## Composition-driven phase diagram

**Figure 1:**
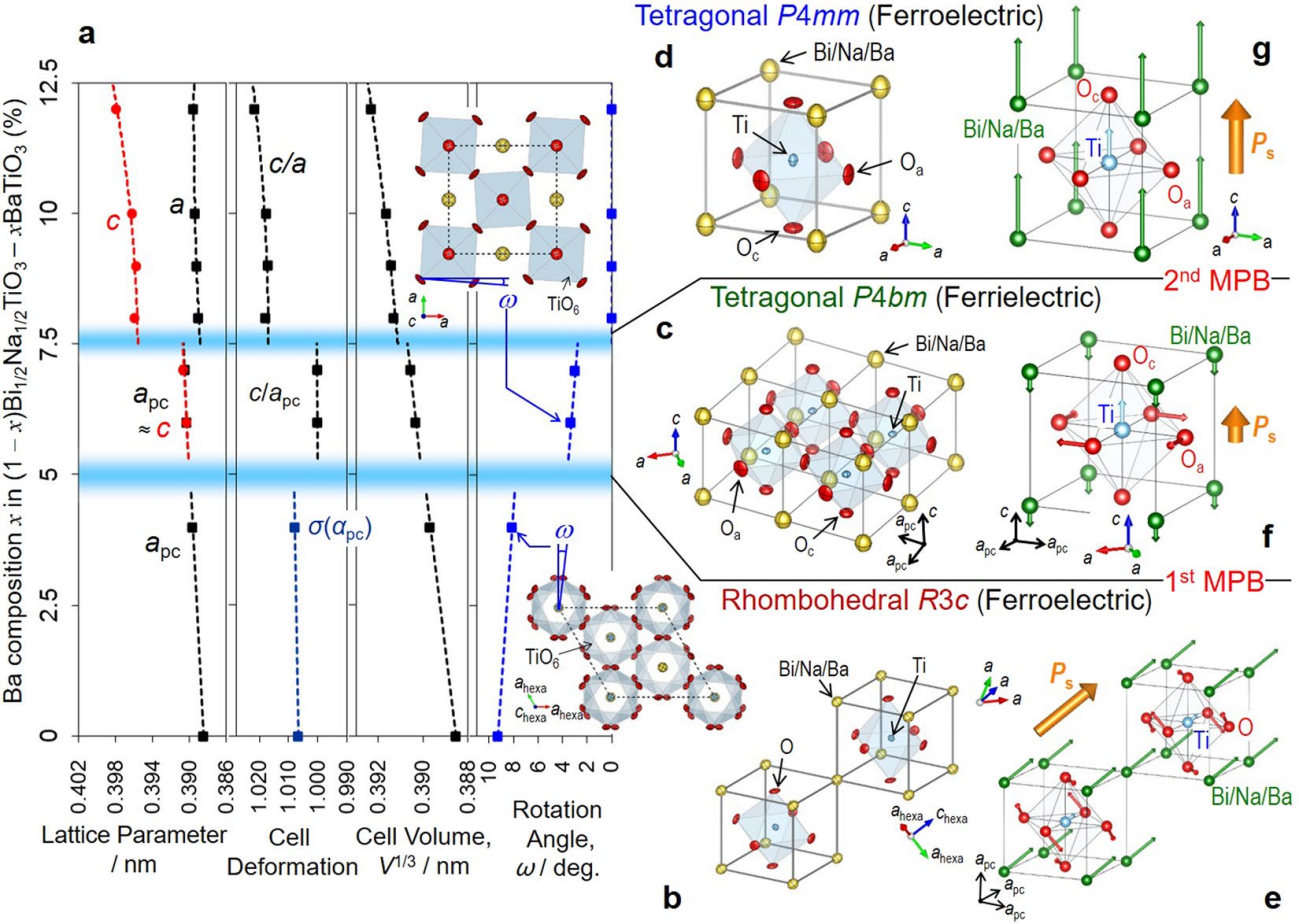
Composition-driven structure evolution. (**a**) Lattice parameters, cell deformation, cell volume (*V*) and rotation angle (*ω*) of TiO_6_ octahedra determined by the high-resolution NPD Rietveld analysis as a function of Ba composition (*x*) in (1 − *x*)Bi_1/2_Na_1/2_TiO_3_–*x*BaTiO_3_. Note that two morphotropic phase boundaries (MPBs) exists, where the tetragonal *P*4*bm* appears as a bridging phase between the ferroelectric phases with rhombohedral *R*3*c* and tetragonal *P*4*mm*. The pseudocubic (pc) parameters are adopted; for the rhombohedral *R*3*c*,* α* and *a* are the angle and parameter, respectively, *σ* represents the cell deformation along [111]_pc_ expressed by $\sigma =\sqrt{({\rm{1}}+{\rm{2cos}}{\alpha }_{{\rm{pc}}})/(1-\,\cos \,{\alpha }_{{\rm{pc}}})}$, and *ω* is the rotation angle about [111]_pc_ (//hexagonal *c* axis, *c*_hexa_); for the tetragonal *P*4*bm*, *a*_pc_ is *a*/√2, and *ω* is the rotation angle about [001]. Tetragonality (*c*/*a*) is plotted as a cell deformation for the *P*4*bm* and *P*4*mm* phases. (**b**–**g**) Crystal structures and atomic displacements; the left panels show the structures with thermal ellipsoids of the constituent atoms; the light panels exhibit the off-center displacements (Δ*z*) along [111]_pc_ (*R*3*c*) and [001] (*P*4*bm* and *P*4*mm*) from the hypothetical paraelectric positions. O_c_ and O_a_ denote the apical and planner O atoms, respectively, for the tetragonal lattices. Note that the tetragonal *P*4*bm* has ferrielectricity arising from up (Ti) and down (the A-site atoms) displacements associated with a small but significant *P*_s_ along [001].

Figure 1 displays the lattice parameters as a function of the Ba composition (*x*) along with the crystal structures and their atomic displacements. The evolution of the neutron powder diffraction (NPD) patterns and the refinement results, along with the structural data, are shown in Supplementary Figs 1 and 2 and Supplementary Tables 2 and 3. In addition to the fundamental *hkl* reflections, superlattice reflections distinct to the constituent phases appear: 1/2{*o o o*} for *x* ≤ 4% and 1/2{*o o e*} for *x* = 6% and 7% (*o* is an odd number, and *e* is an even number). The 1/2{*o o o*} reflection arises from an out-of-phase TiO_6_ rotation about [111]_pc_ (*a*^−^*a*^−^*a*^−^ in Glazer notation^31^) typical for the rhombohedral *R*3*c* while the 1/2{*o o e*} reflection is attributed to an in-phase TiO_6_ rotation about [001] (*a*^0^*a*^0^*c*^+^) of the tetragonal *P*4*bm*^22,25–27,30^. We assign *x* ≥ 8% to the tetragonal *P*4*mm*, as identified by a clear splitting of the 200 and 002 reflections resulting from an apparent tetragonality (*c*/*a*)^32,33^. The variation in the superlattice reflections are consistent with our synchrotron-radiation X-ray diffraction analysis on single crystals^25^, where extrinsic factors such as ferroelectric and/or ferroelastic domain structures play no role.

We note that the two distinct MPBs exist at *x* ~ 5% and ~ 7.5%: the Ba-poor rhombohedral *R*3*c* with a relatively large ferroelectric distortion (*σ*) of ~1%, the intermediate tetragonal *P*4*bm* with an extremely small tetragonality (*c*/*a*_pc_ ≈ 1.00), and the Ba-rich tetragonal *P*4*mm* with a tetragonality (*c*/*a*) of ~1.02. We did not find a phase coexistence around the MPBs reported in the previous reports^26,27,30^. Across the first MPB at *x* ~ 5%, the mode and magnitude of the TiO_6_ rotation change; a rotation angle (*ω*) of the rhombohedral *R*3*c* is as large as ~8 deg., while that of the tetragonal *P*4*bm* is ~3 deg. (Fig. 1a). The polar atomic displacements (Δ*z*) are schematized in Fig. 1e–g and the electric dipole moments (*p* = Δ*z* × *Z*_eff_*) are depicted in Supplementary Fig. 3. The rhombohedral *R*3*c* features a cooperative displacement of the A-site atoms and Ti along the hexagonal *c*_hexa_ axis (//[111]_pc_). We found that the tetragonal *P*4*bm* displays a ferrielectric polar configuration that up and down dipole moments give rise to nonzero *P*_s_. Beyond the second MPB at *x*~7.5%, the tetragonal symmetry changes to *P*4*mm*, where the A-site atoms and Ti exhibit a robust cooperative off-centring along [001]. We think that the maximal piezoelectric properties reported for the ceramics with around *x* = 6–7%^21,23,27,34,35^ are directly related to the ferrielectric-mediated MPBs.

### First morphotropic phase boundary (^1st^ MPB)

Our NPD analysis for the solid solutions provides an averaged structure regarding the A-site atoms but reveals the precise conformations of TiO_6_ octahedra. In the DFT calculations to investigate the 1^st^ MPB for the Bi_1/2_Na_1/2_TiO_3_ cell (Supplementary Fig. 4), the experimentally determined TiO_6_ structure is adopted; the data of *x* = 4% are used for the rhombohedral *R*3*c* and those of *x* = 6% for the tetragonal *P*4*bm*, because an essential local structure of TiO_6_ does not show a significant change, regardless of the Ba composition^36^. The optimization of the positions of Bi and Na under the constraint of the fixed fractional coordinates of TiO_6_ octahedra yields information on the free energy and the chemical bonding in the lattice environments similar to the real crystal.

### Free energy and Bi-O lengths

**Figure 2:**
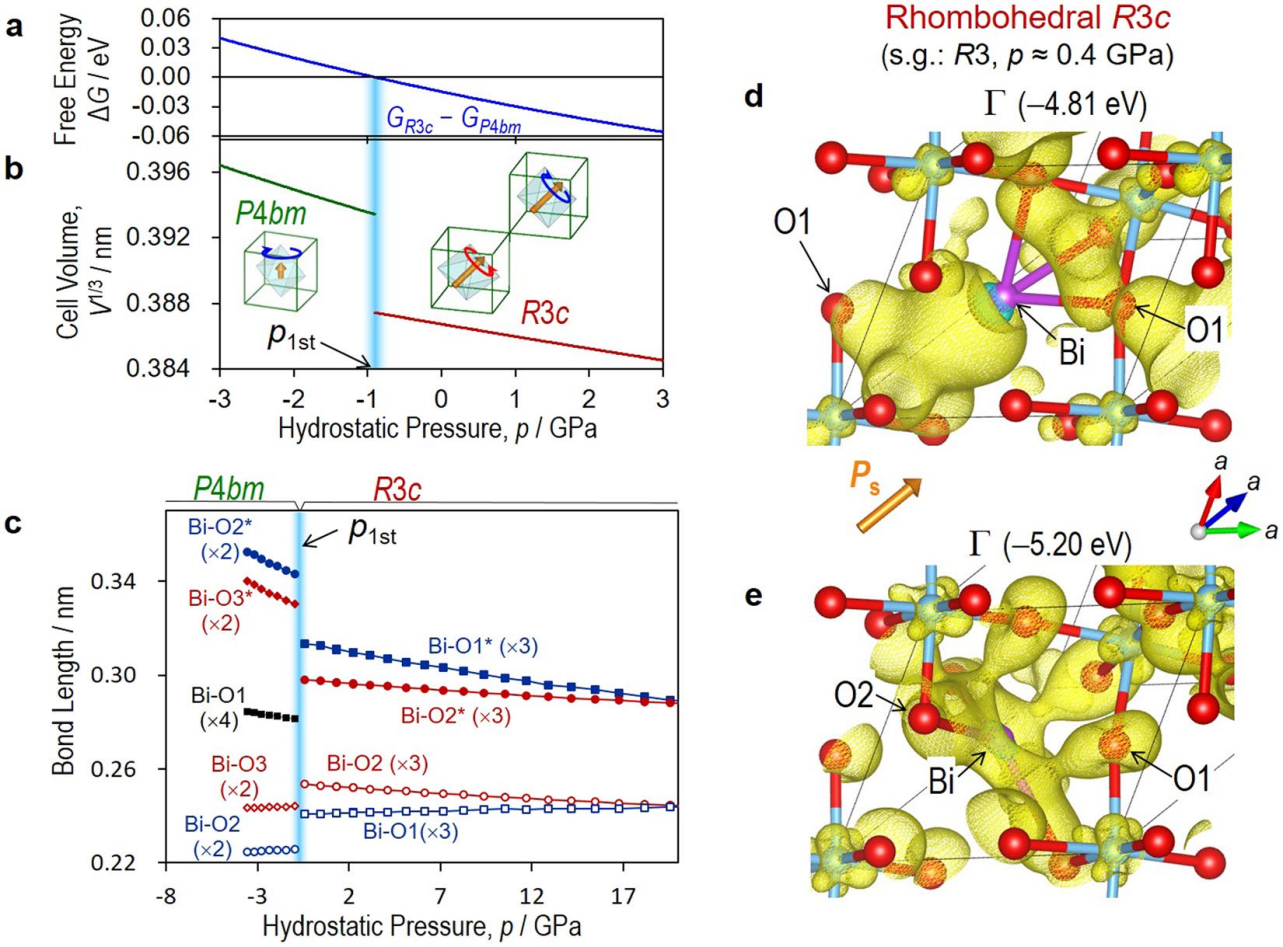
Nature of first morphotropic phase boundary. The Bi_1/2_Na_1/2_TiO_3_ cells (Supplementary Fig. 4) are adopted for the DFT calculations of the rhombohedral *R*3*c* and the tetragonal *P*4*bm*. (**a**) Free-energy difference Δ*G* = *G*_*R*3*c*_ − *G*_*P*4*bm*_ and (**b**), cell volume *V* per ABO_3_ unit cell as a function of pressure *p*. From the fitting analysis of the total energy *U*(*V*) shown in Supplementary Fig. 5a, we obtain *G*_*R*3*c*_ for the *R*3*c* phase and *G*_*P*4*bm*_ for the *P*4*bm* one. The first phase-boundary *p* (*p*_1st_) separating the *R*3*c* and *P*4*bm* phases is present at ~−1 GPa. (**c**) Bi-O bond lengths as a function of *p*, where asterisk (*) denotes longer bond owing to the off-centring of Bi. Wavefunctions of the rhombohedral *R*3*c* at the Γ point whose energy levels are (**d**) −4.81 eV and **e** −5.20 eV, where the optimized structure at *p* ≈ 0.4 GPa is adopted. In (**d**), the bonding interaction between Bi-6*p* and O1-2*p* is seen not only for the short Bi-O1 but also for the long Bi-O1*. In (**e**), the orbital mixing of Bi-6*p* and O-2*p* is seen for the short Bi1-O2, where the interaction of the short Bi1-O1 has a minor role. With decreasing *p*, Bi-O1* and Bi-O2 are lengthened, and thereby these states are lower in energy, leading to a destabilization of the rhombohedral *R*3*c*.

Figure 2 exhibits the relations between free energy *G*, cell volume *V* and pressure *p* (see the total energy *U*(*V*) curves in Supplementary Fig. 5a). Using the fitting parameters in Eq. 1, we obtain *G*_*R*3*c*_ for the rhombohedral *R*3*c* and *G*_*P*4*bm*_ for the tetragonal *P*4*bm* along with their difference, which is expressed as Δ*G* = *G*_*R*3*c*_ − *G*_*P*4*bm*_. We identified the 1^st^ phase-boundary *p* (*p*_1st_) at ~−1 GPa. In the higher-*p* region, Δ*G* becomes negative; the rhombohedral *R*3*c* is stabilized at a smaller *V* (Fig. 2c). In contrast, in the lower-*p* region, Δ*G* becomes positive; the *P*4*bm* phase is lower in free energy at a larger *V*. In the rhombohedral *R*3*c*, e.g., at *p* ~ 0.4 GPa, the bond valence sum (BVS) (Supplementary Fig. 5b) is estimated to be 1.12 for Na and 4.18 for Ti, which are close to their respective formal valences (Na^+^ and Ti^4+^). The BVS of Bi is yet 2.65, which is much smaller than the formal valence (Bi^3+^). With decreasing *p* and beyond *p*_1st_, the BVS of Bi recovers to ~2.73 in the tetragonal *P*4*bm*, whereas those of Na and Ti do not exhibit an anomaly.

The length of Bi-O as a function of *p* (Fig. 2c) provides insight into the 1^st^ MPB. In the centrosymmetric structure, Bi is surrounded by twelve O atoms. In the rhombohedral *R*3c, Bi is displaced along [111]_pc_, leading to four different lengths: the shortest Bi-O1 (×3), followed by Bi-O2 (×3), Bi-O2* (×3) and Bi-O1* (×3), where asterisk (*) denotes a longer bond. Essentially, Bi-O1 is independent of *p* at ~0.240 nm, whose length is in good agreement with the experiments^37^. In contrast, Bi-O2 is lengthened when *p* decreases, leading to a smaller BVS of Bi. In the tetragonal *P*4*bm*, the displacement of Bi along [001] results in three different lengths: the shorter four, the intermediate four (Bi-O1) and the longer four. Moreover, the in-plane TiO_6_ rotation enables Bi to form the shortest Bi-O2 (×2) and the next Bi-O3 (×2). The tetragonal *P*4*bm* accommodates the markedly short Bi-O2 of ~0.225 nm, followed by the comparably short Bi-O2 of ~0.243 nm with respect to Bi-O1 in the rhombohedral *R*3*c*. The in-phase TiO_6_ rotation is accompanied by the short Bi-O2 even though the Δ*z* of Bi is relatively small.

### Electronic origin

We investigate the electronic structures of the rhombohedral *R*3*c* at *p* ≈ 0.4 GPa and the tetragonal *P*4*bm* at *p* ≈ −2.8 GPa (see Supplementary Figs 6 and 7). In the rhombohedral *R*3*c*, the majority of the occupied Bi-6*p* states is higher in energy from −5.6–−4.4 eV at *p* ≈ 9 GPa to −5.2–−4.2 eV at *p* ≈ 0 GPa. The minimum of the valence band is formed by the wave function composed mainly of the *p* states of Bi, O1, and O2 (Fig. 2e); its energy increases from −5.6 eV at *p* ≈ 9 GPa to −5.2 eV at *p* ≈ 0.4 GPa at the Γ point, which is attributed to a lengthened Bi-O2. The next-lowest band is formed by the hybridized orbital of Bi-6*p* and O1-2*p* (Fig. 2d). Even though Bi-O1 remains unchanged, its energy increases from −5.3 eV at *p* ≈ 9 GPa to −4.8 eV *p* ≈ 0.4 GPa at the Γ point because Bi-O1* substantially increases in length. The rhombohedral instability caused by the elongated Bi-O2 and Bi-O1* near *p*_1st_ stems from the shift of the Bi-6*p*-derived states to higher energy. The details of the tetragonal *P*4*bm* are described later.

### Second morphotropic phase boundary (^2nd^ MPB)

Across the 2^nd^ MPB, the phase changes from the tetragonal *P*4*bm* to the tetragonal *P*4*mm*. Because the *P*4*bm* phase has an in-plane TiO_6_ rotation, it is natural to consider that the rotation distortion is suppressed near the 2^nd^ MPB, as observed in the experiments (Fig. 1a). Taking into account the evolution of the TiO_6_ rotation along with the atomic displacements, we performed the structural optimization of the Ba_2/8_Bi_3/8_Na_3/8_TiO_3_ cell (Supplementary Fig. 8) under the constraint of the experimentally determined tetragonality of *c*_pc_/*a*_pc_ = 1.0001 (*x* = 6%) for the *P*4*bm* phase and *c*_pc_/*a*_pc_ = 1.0218 (*x* = 12%) for the *P*4*mm* phase.

### Free energy and reconstruction of Bi-O bonds

**Figure 3:**
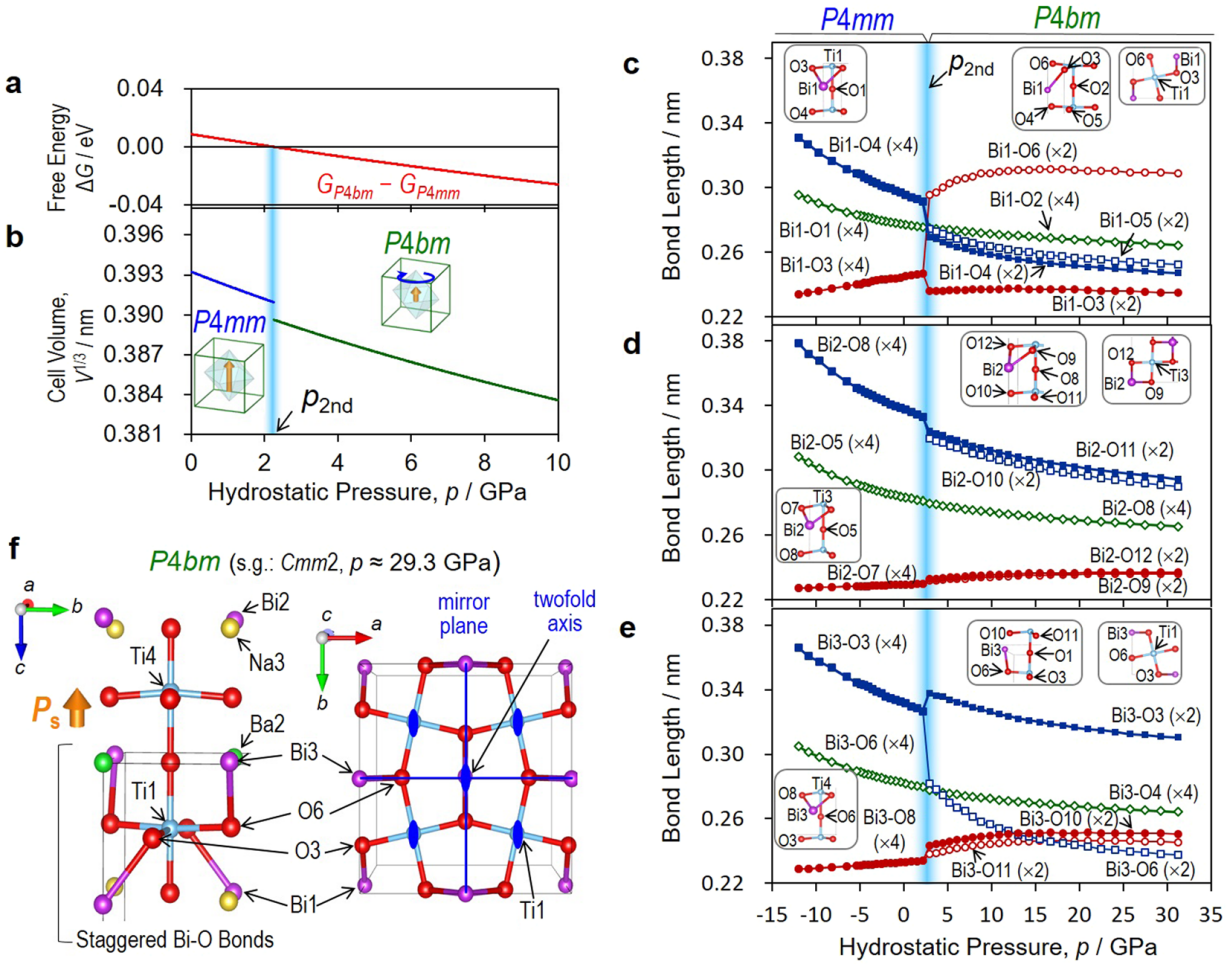
Nature of second morphotropic phase boundary. The Ba_2/8_Bi_3/8_Na_3/8_TiO_3_ cells (Supplementary Fig. 8) are adopted for the DFT calculations of the tetragonal *P*4*bm* and the tetragonal *P*4*mm*. (**a**) Free-energy difference Δ*G* = *G*_*P*4*bm*_ − *G*_*P*4*mm*_ and (**b**), cell volume *V* per ABO_3_ unit cell as a function of pressure *p*. From the fitting analysis of the total energy *U*(*V*) shown in Supplementary Fig. 9a, we obtain *G*_*P*4*bm*_ for the *P*4*bm* phase and *G*_*P*4*mm*_ for the *P*4*mm* one. The second phase-boundary *p* (*p*_2nd_) separating the *P*4*bm* and *P*4*mm* phases is present at ~ ~2 GPa. Bi-O bond lengths as a function of *p* for (**c**) Bi1-O, (**d**) Bi2-O and (**e**) Bi3-O. Note that a reconstruction was found for Bi1-O and Bi3-O at *p*_2nd_, whereas Bi2-O presents a monotonic tendency. (**f**) Staggered Bi-O conformation composed of the short Bi1-O3 and Bi3-O6 bonds in the tetragonal *P*4*bm* leading to a robust in-phase TiO_6_ rotation, where the *P*_s_ vector is parallel to the dipole moment derived from the Bi-Na and Bi-Ba layers.

Figure 3 shows the relation between Δ*G* = *G*_*P*4*bm*_ − *G*_*P*4*mm*_, *V* and *p*, along with the Bi-O lengths, as a function of *p*. The *U*(*V*) curves are presented in Supplementary Fig. 9. We found the 2^nd^ phase-boundary *p* (*p*_2nd_) at ~2 GPa: Δ*G* becomes negative in the higher-*p* region; the *P*4*bm* phase is stabilized at a smaller *V*, whereas Δ*G* becomes positive in the lower-*p* region; and the *P*4*mm* phase emerges at a larger *V*.

Our DFT calculations shed light on the structural origin of the in-phase TiO_6_ rotation. The octahedral rotation angle (*ω*) as a function of *p* is shown in Supplementary Fig. 10. The *ω* of Ti1-O_6_ sandwiched between the Bi-Ba and Bi-Na layers is as large as ~12 deg., where its rotation axis is parallel not only to the dipole moment derived from Na and Ba but also to the *P*_s_ vector. We note that a staggered conformation of Bi-O is formed around Ti1-O_6_ by the shortened Bi1-O3 and Bi3-O6 bonds (Fig. 3f), which stems from its large *ω*. The *ω* of Ti1-O_6_ is significantly reduced when approaching *p*_2nd_, suggesting that the instability of the *P*4*bm* phase is closely related to the suppression of the Ti1-O_6_ rotation. In reality, for the lattice of the solid solution where Bi, Ba, and Na have a quasi-random distribution^28^, we consider the following structural feature: the Ti1-O_6_ unit with the staggered Bi-O bonds drives a coherent in-phase rotation about the polar axis in the entire crystal; thus, the resultant *ω* is averaged to 2–3 deg., as observed in Fig. 1.

Although we did not find a prominent feature in the BVSs as a function of *p* (Supplementary Fig. 11), it is noticeable that Bi-O shows a reconstruction across *p*_2nd_; Bi1-O and Bi3-O display discontinuous changes in length caused by the Ti1-O_6_ rotation, whereas Bi2-O exhibits a monotonic tendency. The off-centring of Bi along [001] in the *P*4*mm* phase results in a short bond with four equivalent O atoms, i.e., Bi1-O3, Bi2-O7, and Bi3-O8, while each of these degenerated bonds splits into two different lengths in the *P*4*bm* phase. Interestingly, Bi1-O exhibits the most striking feature across *p*_2nd_: Bi1-O3 in the *P*4*mm* phase is divided into the shortest Bi1-O3 and the longest Bi1-O6 in the *P*4*bm* phase, which results from the Ti1-O_6_ rotation. Given that *p* decreases and approaches *p*_2nd_, Bi-O6 is substantially shortened, whereas Bi1-O3 remains unchanged. In addition, a decrease in *p* elongates Bi1-O4 and Bi1-O5, which leads to a smaller BVS of Bi1 to 2.54 near *p*_2nd_.

### Electronic origin

**Figure 4:**
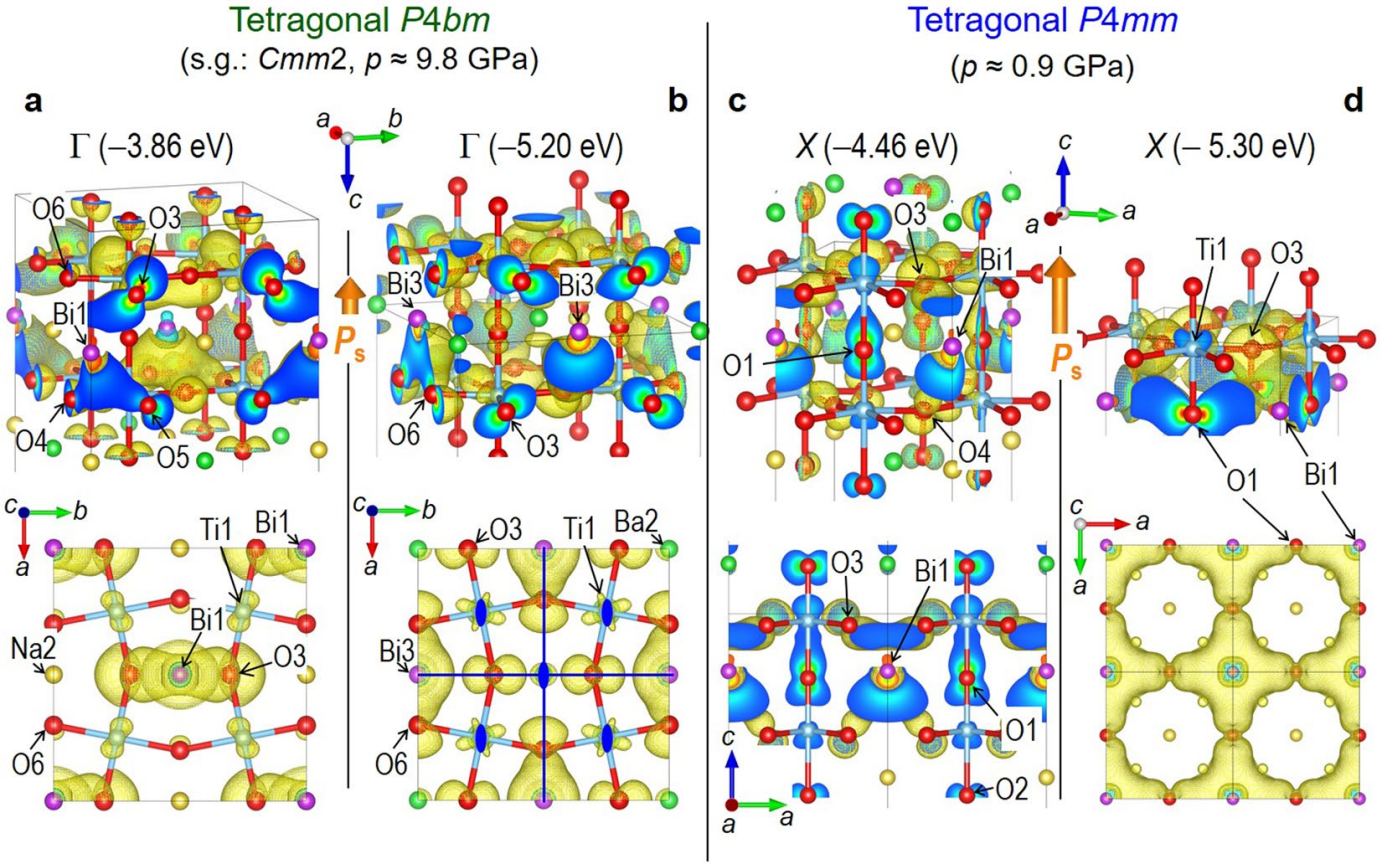
Wave functions. The orbital interactions in the Ba_2/8_Bi_3/8_Na_3/8_TiO_3_ cells (Supplementary Fig. 8) that stabilize polar and rotation distortions: for the tetragonal *P*4*bm* (*p* ≈ 9.8 GPa), the orbitals at the Γ point whose energy levels are (**a**) −3.86 eV and (**b**) −5.20 eV; for the tetragonal *P*4*mm* (*p* ≈ 0.9 GPa), those at (**c**) the *X* point (−4.46 eV) and **d** the *X* point (−5.30 eV). For both phases, the hybridizations of **c** the out-of-plane Bi(6*p*_*z*_)-O(2*p*) and (**d**) the in-plane Bi(6*p*_*x*_ + *p*_*y*_)-O(2*p*) give rise to the bonding states at the bottom of the valence bands, leading to the Bi displacement along [001]. In addition, the *P*4*bm* phase is stabilized by the staggered Bi(6*p*_*z*_)-O(2*p*) hybridizations (**a**,**b**) associated with the short Bi1-O3 and Bi3-O6 bonds, which drives a robust Ti1O_6_ rotation. In (b) (lower panel), solid blue lines representing mirror plane and closed ellipses denoting twofold axis are depicted. These symmetry elements are present also in (a) (lower panel).

First, we describe the relation between the crystal structure and the electronic states of the *P*4*mm* phase (see Supplementary Fig. 12b). Two common features appear in the orbital interactions regarding Bi-6*p*, which constitutes most of the lower part of the valence band. The first is the hybridization in the O-Bi(6*p*_*x*_ + *p*_*y*_)-O-Ti unit, e.g., Bi1 has low-lying 6*p*_*x*_ + *p*_*y*_ states at ~−5.3 eV (Bi1-6*p*_*z*_ is higher in energy at ~−4.5 eV). In the O1-Bi1-O3-Ti1 unit, as seen in the wavefunction at the *X* point (Fig. 4d), Bi1-6*p*_*x*_ + *p*_*y*_ is mixed not only with the nearest O3-2*p* but also with the adjacent Ti1-3*d*, in addition to a significant contribution from the next-nearest O1-2*p*. A similar feature is seen also in the O5-Bi2-O7-Ti3 unit, which constitutes the minimum valence band. The second is the hybridization in the O-Bi(6*p*_*z*_)-O unit. In the O1-Bi1-O3 unit, Bi1-6*p*_*z*_ forms the bonding states with O1-2*p* and O3-2*p*, as found in the wavefunction at the *X* point (Fig. 4c). Because the displacements of Bi2 and Bi3 along [001] are much larger than that of Bi1 (Bi2-O7 and Bi3-O8 become short), their bonding orbitals in the O-Bi(6*p*_*z*_)-O units are lower in energy, as seen in the density of states (DOS).

In addition to the orbital interactions described above, distinct hybridizations emerge in the *P*4*bm* phase, where Bi-6*p*_*z*_ plays a crucial role. According to the wave function at the Γ point in Fig. 4a, Bi1-6*p*_*z*_ is hybridized with O3-2*p*, in which O4-2*p* and O5-2*p* have a minor contribution whereas O6 does not participate. The energy of this state at *p* ~ 2.2 GPa is −3.86 eV and that of the other Bi1-6*p*_*z*_-derived state is −4.67 eV at the *Y* point (Supplementary Fig. 13). An increase in *p* lowers these energies by 0.1–0.2 eV, which is partly attributed to the lower *G*_*P*4*bm*_. In addition, the orbital mixing of Bi3-6*p*_*z*_ and O6-2*p* (Fig. 4b) leads to the short Bi3-O6, which contributes to the large *ω* of Ti1-O_6_. We found that the in-phase TiO_6_ rotation stabilizing the *P*4*bm* phase originates from the staggered Bi-O bonds (Fig. 3f) derived from the Bi-6*p*_*z*_ and O-2*p* interaction.

## Discussion

We demonstrate the ferrielectric-mediated MPBs where the tetragonal *P*4*bm* appears as a bridging phase. The ferrielectric *P*4*bm* has two order parameters: polarization (*p*) and octahedral rotation (*ω*); their coupling energy can be expressed as *G*_c_ = *κ*(*p*^2^*ω*^2^ + *p*^4^*ω*^2^), where *κ* is constant^25^. Given that *G*_c_ exceeds a certain threshold, the energy landscape has an extremely flat valley, along which an application of electric field (*E*) displaces the *P*4*bm* phase substantially from the ground state. It is worth noting that the single crystal in the *P*4*bm* phase displays an intrinsic piezoelectric strain constant (*d*_33_) of as high as 1,000 pm/V (ref.^25^), which is much larger than that of conventional Pb(Zr, Ti)O_3_ ceramics^4^. Moreover, the *P*4*bm* phase undergoes an *E*-induced phase transition to the *P*4*mm* phase even at a small *E* of 20 kV/cm, which is accompanied by an extremely large strain increase by ∼0.6%. By exploiting the intrinsic response superimposed on the field-induced phase transition, we could achieve high-performance Bi-based piezoelectric materials.

The ferrielectric *P*4*bm* is expected to emerge in the solid solution with multiferroic BiFeO_3_, since BiFeO_3_ has the same symmetry with Bi_1/2_Na_1/2_TiO_3_ (rhombohedral *R*3*c* with *a*^−^*a*^−^*a*^−^)^38^, raising the possibility of a novel multiferroic nature with ferrielectric polarization and (anti)ferromagnetic order. Moreover, an electric control of magnetization could be dramatically enhanced when an *E*-induced ferri- to ferroelectric transition takes place, because ∂*P*/∂*E* of the phase transition is extraordinary, ∂*P*/∂*E* > 10,000*ε*_0_ (*ε*_0_ is the permittivity of vacuum), which is several orders of magnitude larger than that of the conventional ferroelectrics^39^. We could design other (multi)ferroic materials as well based on the staggered chemical bonds at an appropriate chemical pressure, here that we have demonstrated the existence and electronic origin of the ferrielectric-mediated MPBs in Bi-based polar perovskites.

## Methods

### Sample preparation

Powders of (1 − *x*)Bi_1/2_Na_1/2_TiO_3_–*x*BaTiO_3_ (*x* = 0, 4%, 6%, 7%, 8%, 9%, 10%, and 12%) were prepared by solid-state reaction of the raw materials of Bi_2_O_3_ (99.99%), TiO_2_ (99.99%), Na_2_CO_3_ (99.99%), and BaCO_3_ (99.99%). These starting materials were thoroughly mixed by ball milling using beads as small as 100 μm and then calcined at 1,223 K for 4 h. The obtained powders were crushed by ball milling and then calcined again at 1,423 K for 4 h in order to achieve a homogeneous solid solution.

### Neutron powder diffraction analysis

Time of flight (TOF) NPD data were collected using a high-resolution neutron powder diffractometer iMateria (BL20)^40^ at Japan Proton Accelerator Research Complex (J-PARC). Using the collected data in the *d* range of 0.05 < *d*/nm < 0.25 with a resolution Δ*d*/*d* = 0.16%, the crystal structure was refined by the Rietveld method using a computer software Z-Rietveld^41^. When required, the pseudocubic (pc) notation is adopted to denote the Miller indices (*hkl*) and the crystal orientation. Recent studies^24,29,37,42,43^ indicate that BNT-BT with a low Ba composition (*x*) at room temperature belongs to space group *Cc* with a small monoclinic distortion from rhombohedral *R*3*c* structure. Since the monoclinic distortion is less than 0.1% and we focus on the marked change in the crystal structure across phase boundaries, we regard the room-temperature phase at a small *x* as rhombohedral *R*3*c* for simplicity throughout this paper.

### *Ab initio* Density functional theory (DFT) calculations

DFT calculations were performed via the generalized gradient approximation^44^ with a plane wave basis set. The projector-augmented wave method^45^ was applied by the Vienna *ab initio* simulation package (VASP)^46^. We employed the gradient-corrected exchange-correlation functional of the Perdew-Burke-Ernzerhof revised for solids (PBEsol)^47^ and a plane-wave cut-off energy of 520 eV. The adopted mesh size of the *k*-point sampling grid was less than 5 nm^‒1^ for structural optimization, 2.5 nm^−1^ for density-functional perturbation theory (DFPT) calculations, 1 nm^−1^ for density of states (DOS) and band-structure calculations.

To obtain the Born effective charges, the atomic positions of Bi and Na were optimized in the (Bi_1/2_Na_1/2_TiO_3_)_6_ cells with an A-site ordering (described below) under the constraints of the fixed TiO_6_ octahedral structures determined by the NPD analysis. Similarly, that of Ba in the BaTiO_3_ cells were optimized in the experimental cell size with the fixed octahedral configurations. Then, we performed the DFPT calculations and obtained the Born effective charges of all the constituent atoms. Adopting a weighted average (mol %) of the Born effective charges (*Z*_eff_*) obtained in their respective Bi_1/2_Na_1/2_TiO_3_ and BaTiO_3_ cells, we estimated the averaged *Z*_eff_* of each atom in the solid solutions, as listed in Supplementary Table 4.

To study the nature of the composition-driven phase boundaries, we have to take into account the following two factors: the arrangement of the A-site atoms and the expansion of the cell volume. For Ba-poor compositions, it is reasonable to adopt the supercell of Bi_1/2_Na_1/2_TiO_3_ according to the experimentally determined crystal structure. In contrast, for Ba-rich compositions, it is necessary to consider the supercell that involves Ba because the tetragonal *P*4*mm* is the feature of BaTiO_3_. Therefore, we performed the DFT calculations to investigate the 1^st^ phase boundary for the (Bi_1/2_Na_1/2_TiO_3_)_6_ cell and the 2^nd^ phase boundary for the (Ba_2/8_Bi_3/8_Na_3/8_TiO_3_)_8_ cell. We confirmed that the influence of partial Ba occupation on the A site can be regarded as an expansion of unit cell volume in our calculations, the details of which are described in Supplementary Fig. 14.

Gröting *et al*.^48–50^ have reported that the A-site arrangement largely influences the phase stability, which is also confirmed in our preliminary calculations. Through comparing several A-site atomic arrangements for investigating how the stable phase varies as experimentally observed, we adopted the rock-salt A-site ordering^25,51^ that provides a reasonable structure for the *P*4*bm* phase having a small *P*_s_ compared with the *P*4*mm* phase^25^. The arrangement of the A-site atoms lowers the symmetry, e.g., the Bi and Na ordering in the rhombohedral changes space group from *R*3*c* to *R*3. For simplicity, the higher symmetry is used to denote the space group throughout this paper.

We calculated the total energy (*U*) per ABO_3_ unit cell as a function of the cell volume (*V*) and then analyzed by the Murnaghan equation of state^52^:

1 $$U(V)={E}_{0}+\frac{{B}_{0}V}{{B^{\prime} }_{0}}[\frac{{({V}_{0}/V)}^{{B^{\prime} }_{0}}}{{{B}_{0}}^{^{\prime} }-1}+1]-\frac{{B}_{0}{V}_{0}}{{B^{\prime} }_{0}-1},$$

where *E*_0_, *B*_0_, *B*_0_′, and *V*_0_ are the total energy, the bulk modulus and its first derivative with respect to the hydrostatic pressure (*p*) and *V* at *p* = 0. Since the free energy (*G*) is expressed as *G* = *U* + *pV*, we can obtain the relation between *G* and *p* using the fitting parameters in Eq. 1.

## Supplementary information

Supplementary Information

## Data Availability

The data that support the findings of this study are available upon request from the corresponding authors.
