# Supplementary material for: Ferrielectric-mediated morphotropic phase boundaries in Bi-based polar perovskites
Source: Sci Rep. 2019 Mar 11;9:4087. doi: 10.1038/s41598-019-40724-1 (PMC6411732; doi:10.1038/s41598-019-40724-1)
Supplement: Supplementary file 1 — Supplementary Information [file 41598_2019_40724_MOESM1_ESM.pdf]

Ferrielectric-mediated morphotropic phase boundaries in Bi-based polar perovskites

Yuuki Kitanaka, Masaru Miyayama, and Yuji Noguchi*,

School of Engineering, The University of Tokyo, 7-3-1 Hongo, Bunkyo-ku, Tokyo 113-856, Japan

*Address correspondence to ynoguchi@fmat.t.u-tokyo.ac.jp and yuji19700126@gmail.com.

TABLE OF CONTENTS

➢ Supplementary Figures

1. Evolution of the neutron powder diffraction patterns
2. Results of the Rietveld refinements
3. Polar atomic displacements and electric dipole moments
4. $Bi_{1/2}Na_{1/2}TiO_3$ cells
5. Total energy (*U*)-cell volume (*V*) relation and bond valence sum (BVS) across the first phase boundary
6. Electronic structures of the $Bi_{1/2}Na_{1/2}TiO_3$ cells
7. Wave function of the tetragonal *P*4*bm* for the $Bi_{1/2}Na_{1/2}TiO_3$ cell
8. $Ba_{2/8}Bi_{3/8}Na_{3/8}TiO_3$ cells
9. *U*(*V*) curves across the second phase boundary
10. Rotation angle (ω) of TiO_6 octahedra in the vicinity of the second phase boundary
11. Bond Valence Sum (BVS) across the second phase boundary
12. Electronic structures for the tetragonal $Ba_{2/8}Bi_{3/8}Na_{3/8}TiO_3$ cells
13. Wavefunction of the tetragonal *P*4*bm* for the $Ba_{2/8}Bi_{3/8}Na_{3/8}TiO_3$ cells
14. Density of states (DOS) of Ba for the *P*4*bm* and *P*4*mm* phases

➢ Supplementary Tables

1. Reported results of the structural analysis in the $(1-x)Bi_{1/2}Na_{1/2}TiO_3$–$x BaTiO_3$ system
2. Crystallographic data of $(1–x)Bi_{1/2}Na_{1/2}TiO_3$–$x BaTiO_3$
3. Fractional coordinates and equivalent anisotropic displacement parameters
4. Born effective charges (Z_{eff}*) obtained by the DFPT calculations
5. Comparison in polarization properties for the *R*3*c*, *P*4*bm*, and *P*4*mm* phases

➢ References in Supplementary Information

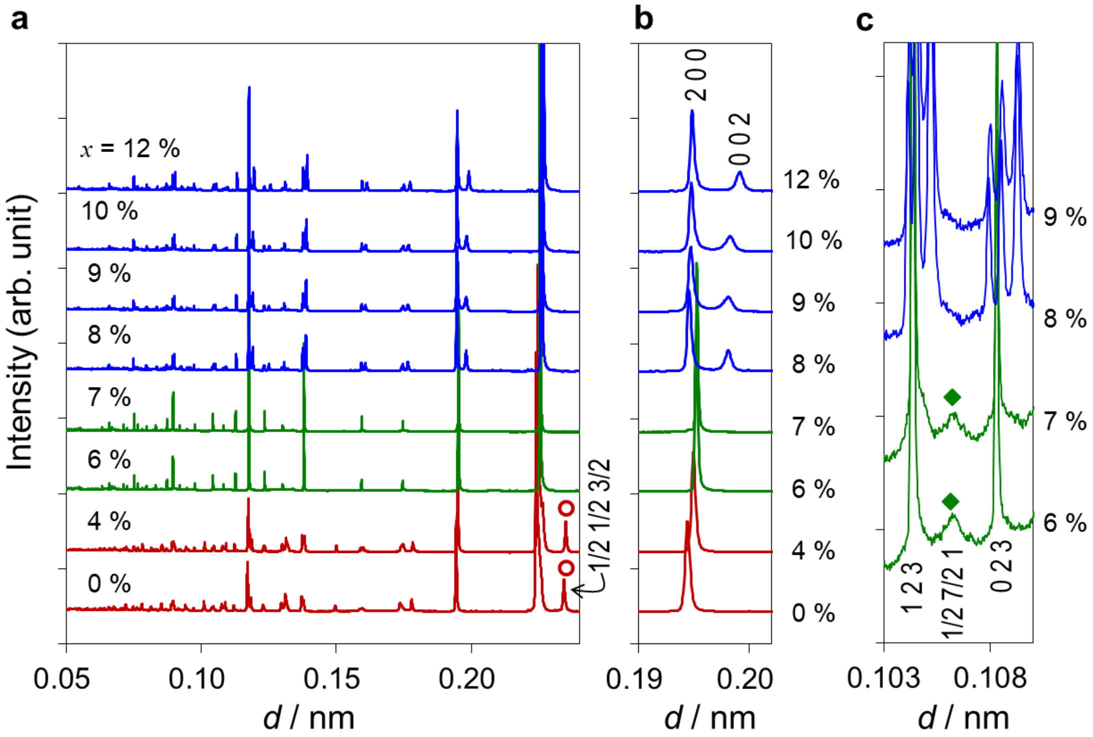

Supplementary Fig. 1 | Evolution of the neutron powder diffraction patterns. The sample composition is expressed as $(1–x)Bi_{1/2}Na_{1/2}TiO_3–xBaTiO_3$, where x denotes the Ba content on the A-site. **a**, the overall feature, **b**, the pseudocubic 200 reflection and **c**, the superlattice reflection observed in the intermediate phase. In the range of $0 \leq x \leq 4$ %, the 1/2{*o o o*} superlattice reflection arising from the $a^-a^-a^-$ octahedral rotation peculiar to the rhombohedral *R*3*c* appears, while the 1/2{*o o e*} one resulting from the $a^0a^0c^-$ rotation distinct to the tetragonal *P*4*bm* arises for $x = 6$ % and 7 % (*o* is an odd number and *e* is an even number). At above $x = 8$ %, the data are well traced by the tetragonal *P*4*mm*, and a tetragonal strain (~2 %) results in a clear splitting of the 200 and 002 peaks (**b**).

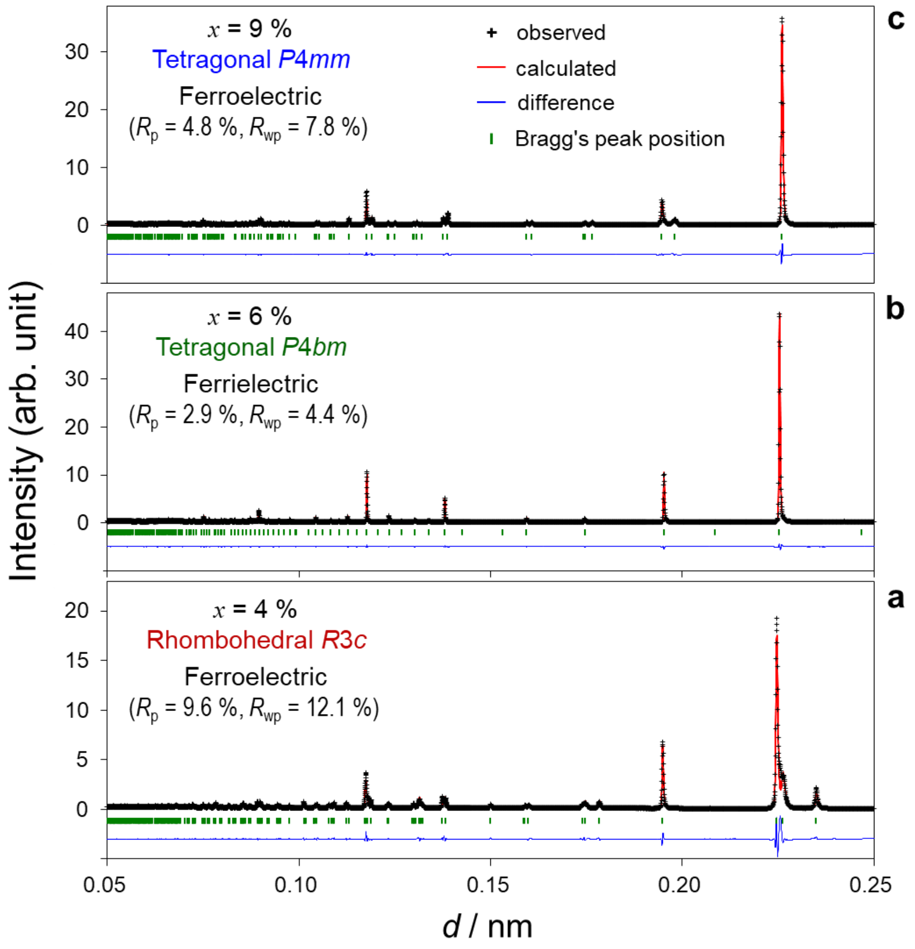

Supplementary Fig. 2 | Results of the Rietveld refinements. The neutron powder diffraction patterns of $(1–x)Bi_{1/2}Na_{1/2}TiO_3–xBaTiO_3$ along with the fitting results for **a** $x = 4$ %, **b** $x = 6$ % and **c** $x = 9$ %. A nearly perfect fitting is achieved for the intensity data for **b** $x = 6$ % and **c** $x = 9$ % by the structures in space group tetragonal *P*4*bm* and tetragonal *P*4*mm*, respectively, and the resultant *R* factors are sufficiently small. The data of **a** $x = 4$ % are well explained by the structural model with rhombohedral *R*3*c*, whereas their *R* factors are relatively large. The model structure with monoclinic *Cc* did not improve the result. The tetragonal *P*4*bm* and *P*4*mm* have a group-subgroup relation, and the phase transition between them is explained by group theory. In contrast, neither rhombohedral *R*3*c* nor monoclinic *Cc* is a subgroup of tetragonal *P*4*bm*. Because the $x = 4$ % sample indeed has an out-of-phase TiO_6 rotation ($a^-a^-a^-$) and the details of the minor structural distortions along with the symmetry relation are less critical, we assign the phase with $x \leq 4$ % as rhombohedral *R*3*c* for simplicity throughout this paper.

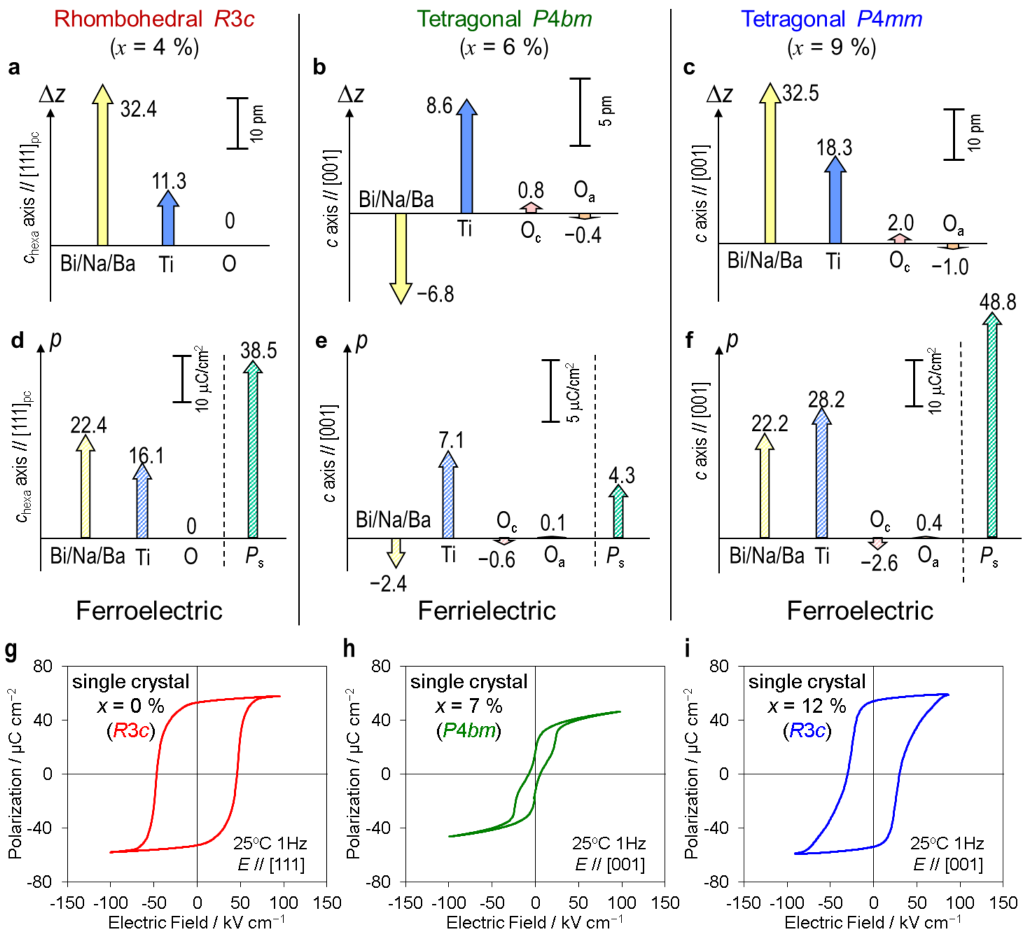

Supplementary Fig. 3 | Polar atomic displacements and electric dipole moments. The displacements along the polar directions (Δ*z*; Fig. **a–c**) from the hypothetical paraelectric positions are estimated from the structural data. The electric dipole moments (*p*; Fig. **d–f**) of the constituent atoms are obtained by $p = \Delta z \times Z_{\mathrm{eff}}^*$. The Born effective charges (Z^*) of Ti and O are adopted for their Z_{eff}^*, while those of the A-site atoms are used to estimate Z_{eff}^*, the weighted average (mol %) of Z^* (see Supplementary Table 4). For the rhombohedral *R*3*c*, the Δ*z* of the A-site atoms is much greater than that of Ti, whereas their *p* values are comparable, leading to a spontaneous polarization (P_s) of 38.5 μC/cm². This estimated P_s is somewhat smaller than that observed for $Bi_{1/2}Na_{1/2}TiO_3$ single crystals (Fig. **g**, 55 μC/cm²)[1,2], partly because of a rhombohedral instability owing to the composition (*x* = 4 %) in the vicinity of the first MPB. Note that the tetragonal *P*4*bm* existing as an intermediate phase not only displays a ferrielectric polar configuration composed of up and down dipole moments but also features an in-phase TiO_6 rotation. The Δ*z* of the A-site atoms leads to $p_A = -2.4$ μC/cm², while that of Ti results in $p_B = -7.1$ μC/cm². This ferrielectric configuration delivers a P_s of 4.3 μC/cm², which agrees well with the measured P_s for the *P*4*bm* phase of the single crystals with x = 7 % (Fig. **h**)[3]. The tetragonal *P*4*mm* has a large P_s of 49 μC/cm², which is consistent with the value observed for the single crystal with *x* = 12 % (Fig. **i**)[4]. The comparison in polarization properties are summarized in Supplementary Table 5.

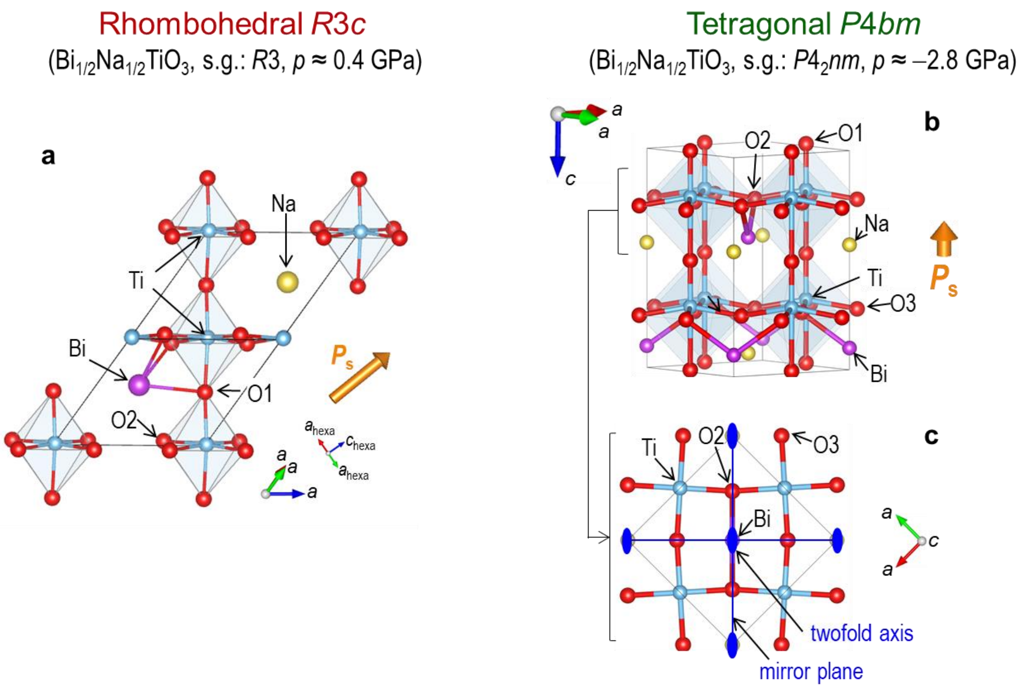

Supplementary Fig. 4 | $Bi_{1/2}Na_{1/2}TiO_3$ cells. For investigating the first MPB by the DFT calculations, we adopt the $Bi_{1/2}Na_{1/2}TiO_3$ cells with a rock-salt-like A-site ordering. The arrangement of Bi and Na on the A site lowers rhombohedral symmetry from *R*3*c* to *R*3 and tetragonal symmetry from *P*4*bm* to $P4_2nm$. We use the higher symmetry to denote these cells along with their DFT results throughout this paper. Owing to the symmetry constraint in **a** the rhombohedral, Bi is displaced along the threefold axis (// $[111]_{pc}$) and thereby constitutes a short bond with three equivalent O atoms. For the tetragonal *P*4*bm* (**b**, **c**), a displacement of Bi along [001] is accommodated, which allows Bi to form a bonding with adjacent four O atoms. Furthermore, the in-plane rotation of TiO_6 about [001] gives rise to the two shortest Bi-O2 bonds. In **c**, solid blue lines represent mirror plane and closed ellipses denotes twofold axis.

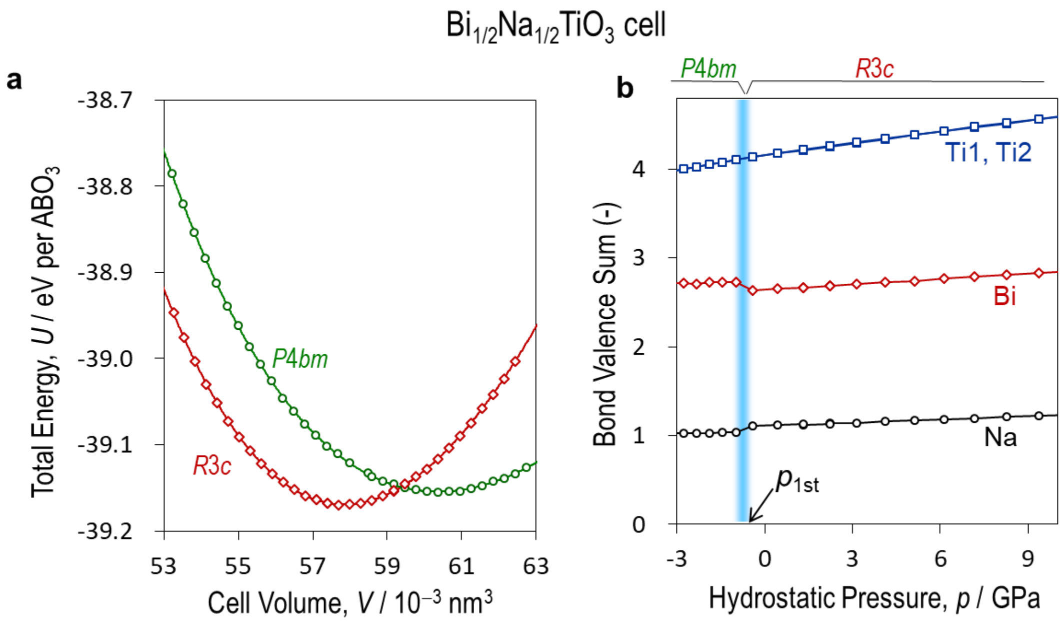

Supplementary Fig. 5 | Total energy (*U*)-cell volume (*V*) relation and bond valence sum (BVS) across the first phase boundary. The *U*(*V*) curves per ABO_3 unit cell and BVS as a function of *p* are obtained by the DFT calculations for the $Bi_{1/2}Na_{1/2}TiO_3$ cells. Data points represent the *U* obtained for the optimized structure at each of the fixed *V*, and the solid lines show the fitting results using the Murnaghan equation of state.

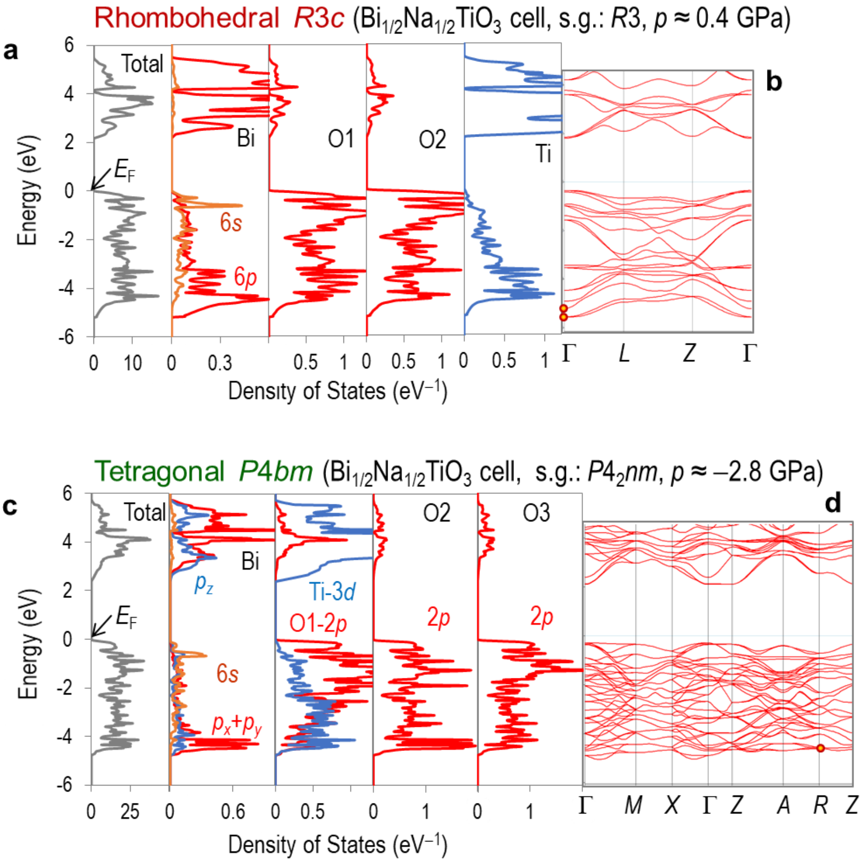

Supplementary Fig. 6 | Electronic structures of the $Bi_{1/2}Na_{1/2}TiO_3$ cells. The optimized structures of the rhombohedral *R*3*c* at $p \approx 0.4$ GPa and the tetragonal *P*4*bm* at $p \approx -2.8$ GPa are selected as their representatives; for the rhombohedral *R*3*c*, **a** the total and partial density of states (DOS) and **b** the electronic band structure are shown, where the vertical energy level is the same; for the tetragonal *P*4*bm*, **c** the total and partial DOS and **d** the electronic band structure are exhibited in a similar manner. E_F is the Fermi energy, which is defined as that of the valence band maximum (VBM). The VBM is formed mainly by O-2*p* while the conduction band minimum (CBM) is constructed by Bi-6*p* and Ti-3*d*. The hybridization of Ti-3*d* and O-2*p* leads to a DOS in the valence band, which contributes to the off-centring of Ti (ref. [5]). Note that Bi-6*p* is mixed with O-2*p* and thereby has a marked DOS in the valence band. In the rhombohedral *R*3*c*, the total amount of DOS of Bi-6*p* in the valence band remains unchanged when *p* changes; the partial charge of Bi-6*p* (~0.76) is not dependent on *p*. In the tetragonal *P*4*bm*, a considerable DOS of Bi-6*p* is seen in the low energy range of –4.8 – –4.1 eV, which is consistent with the larger BVS of Bi (2.71). A hybridization with O-2*p* results in a high DOS of Bi-6p_x + p_y compared with p_z. The lowest states of the valence band are formed primarily by Bi6-p_x + p_y and O2-2*p* + O3-2*p*.

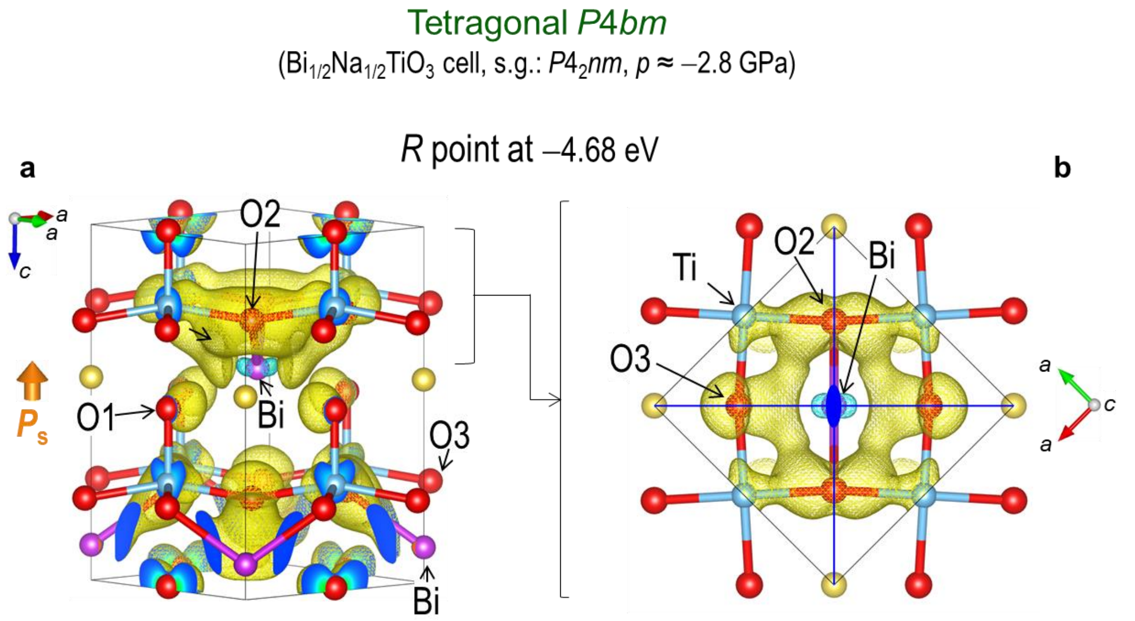

Supplementary Fig. 7 | Wave function of the tetragonal *P4bm* for the $Bi_{1/2}Na_{1/2}TiO_3$ cell. The hybridized orbital between Bi6-p_x + p_y and O2-2*p* + O3-2*p* at the *R* point (4.68 eV), which constitutes the lowest degenerated states of the valence band; **a**, the stereoscopic image and **b**, that projected along the *c* axis. This orbital interaction promotes a displacement of Bi along [001]. The stabilization of the tetragonal *P*4*bm* stems from the short covalent Bi-O arising from the bonding interaction between Bi-6*p* and O2-2*p*.

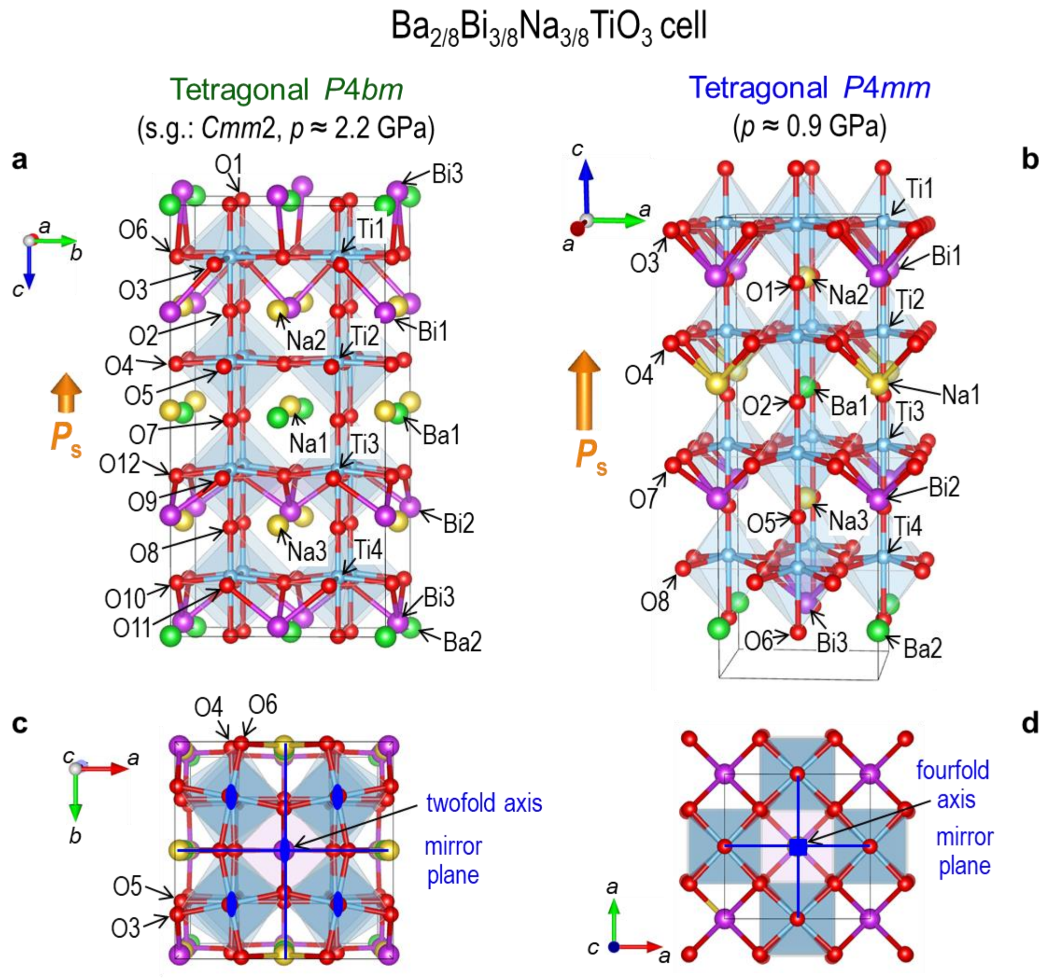

Supplementary Fig. 8 | $Ba_{2/8}Bi_{3/8}Na_{3/8}TiO_3$ cells. For investigating the second MPB by the DFT calculations, we employ the $Ba_{2/8}Bi_{3/8}Na_{3/8}TiO_3$ cells with a rock-salt-like A-site ordering. The arrangement of Bi, Na and Ba on the A site lowers symmetry from tetragonal *P*4*bm* to orthorhombic *Cmm*2 (**a**, **b**), while the symmetry of the *P*4*mm* cell (**c**, **d**) remains preserved. We use the term 'tetragonal *P*4*bm*' instead of orthorhombic *Cmm*2 to denote this cell and the resultant DFT results throughout this paper. The structural optimizations of the atomic positions and cell shape were performed under the constraint of the fixed pseudocubic tetragonalities determined by the experiments. For both cells, all atoms can have an arbitrary fractional coordinate of *z*. The *P*4*bm* cell allows TiO_6 octahedra to have an in-phase rotation, which is ascribed to an additional degree of freedom for the planner O atoms to move in the *a*-*b* plane. In **c** and **d**, solid blue lines represent mirror plane. Closed ellipse and square denote twofold and fourfold axes, respectively.

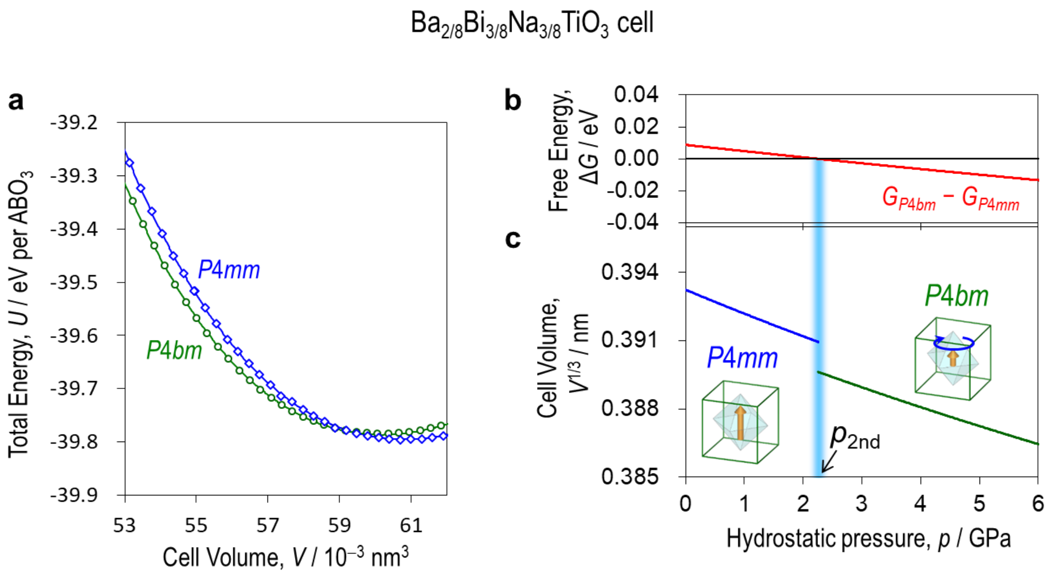

Supplementary Fig. 9 | *U*(*V*) curves across the second phase boundary. The total energy as a function of the ABO_3 cell volume $U(V)$ is obtained by the DFT calculations for the $Ba_{2/8}Bi_{3/8}Na_{3/8}TiO_3$ cells. Data points represent the U obtained by the structural optimization at each of the fixed V, and the solid lines show the fitting results using the Murnaghan equation of state.

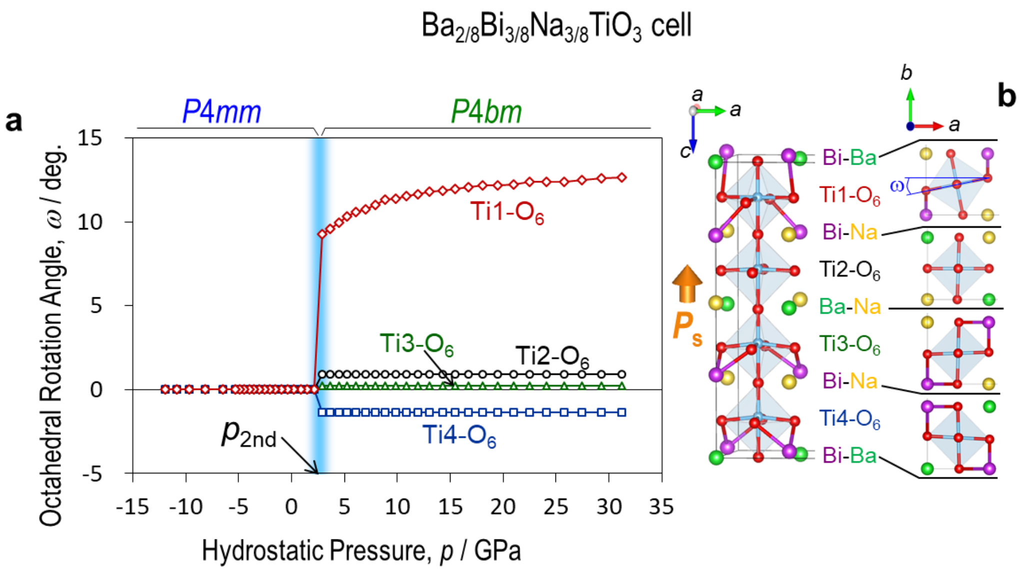

Supplementary Fig. 10 | Rotation angle (ω) of TiO_6 octahedra in the vicinity of the second phase boundary. The angle (ω) of the in-phase TiO_6 rotation is estimated from the optimized *P*4*bm* structures of the $Ba_{2/8}Bi_{3/8}Na_{3/8}TiO_3$ cell. There exist four kinds of TiO_6 octahedra that are accommodated in the different cuboids because of the arrangement of the A-site atoms and the presence of P_s along [001] (the opposite to the *c* axis). The octahedra sandwiched between the Bi-Ba and Bi-Na layers (Ti1-O_6 and Ti4-O_6) exhibit an apparent rotation whereas those between the Bi-Na and Ba-Na layers (Ti2-O_6 and Ti3-O_6) have an almost zero ω. The hypothetical paraelectric structure possesses the mirror plane normal to the *c* axis, and then Ti1-O_6 and Ti4-O_6 between the Bi-Na and Bi-Ba layers have the same structural environment. In the polar lattice, where the small but significant P_s is present along [001], the dipole moment derived from the Bi-Na and Bi-Ba layers adjacent to Ti1-O_6 is parallel to the P_s vector, while that to Ti4-O_6 is opposite to it. This parallel configuration is ascribed to the large ω of the Ti1-O_6 octahedron.

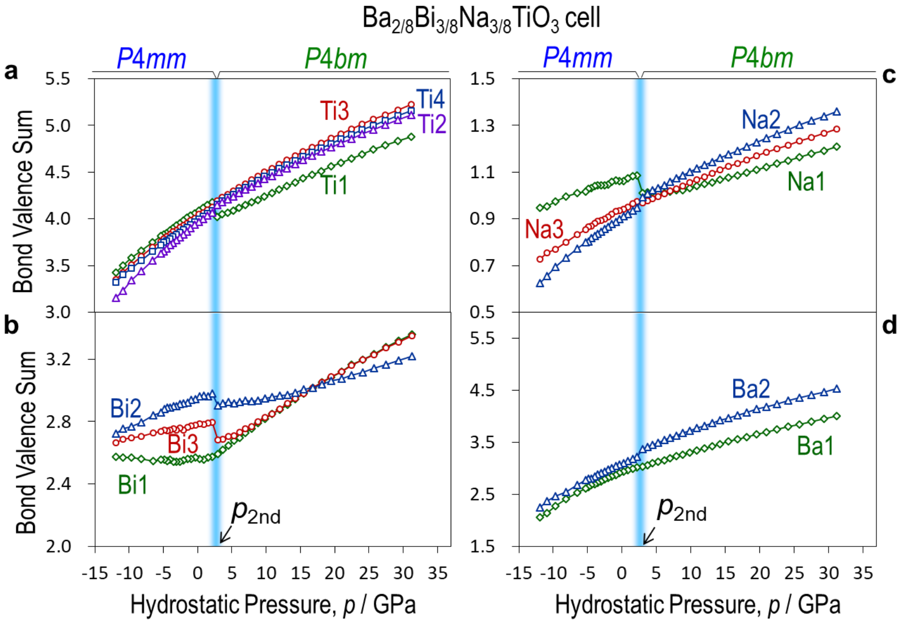

Supplementary Fig. 11 | Bond Valence Sum (BVS) across the second phase boundary. The BVSs as a function of *p* are estimated from the optimized structures obtained by the DFT calculations for the $Ba_{2/8}Bi_{3/8}Na_{3/8}TiO_3$ cells. All the atoms have a downward tendency of BVS with decreasing *p*. Near the second phase-boundary *p* (p_{2nd}), the BVSs of Na and Ti are in good agreement with their respective formal valences (Na^+ and Ti^{4+}), while that of Ba is larger than the formal valence (Ba^{2+}) but is comparable to 2.73 for tetragonal $BaTiO_3$ (ref. [6]). The BVS of Bi2 is close to the formal valence (Bi^{3+}), whereas those of Bi1 and Bi3 are smaller and exhibit a reconstruction of their bonds with O atoms across p_{2nd} (see Fig. 3).

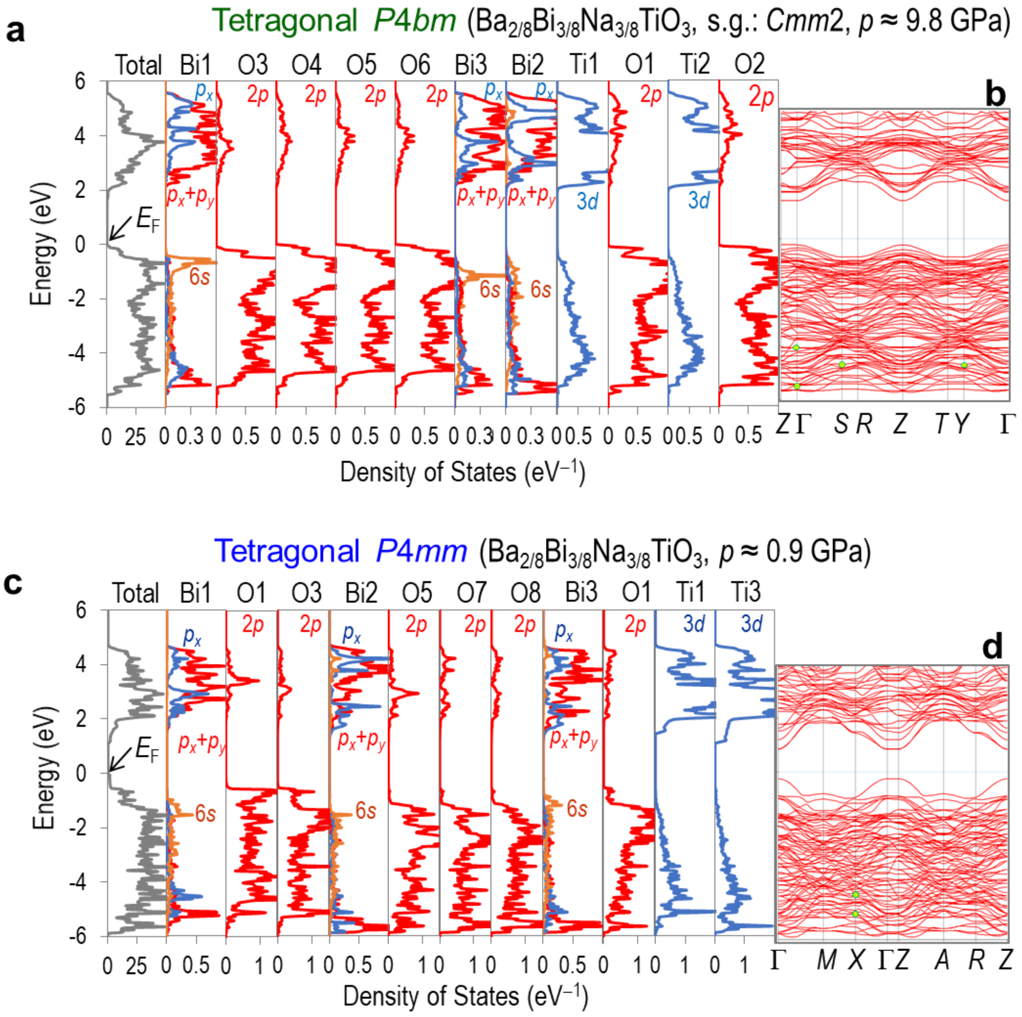

Supplementary Fig. 12 | Electronic structures for the tetragonal $Ba_{2/8}Bi_{3/8}Na_{3/8}TiO_3$ cells. The optimized structures of the *P*4*bm* phase at $p \approx 2.2$ GPa and the *P*4*mm* phase at $p \approx 0.9$ GPa are selected as their representatives; for the *P*4*bm* phase, **a** the total and partial density of states (DOS) and **b** the electronic band structure are shown, where the vertical energy level is the same; for the *P*4*mm* phase, **c** the total and partial DOS and **d** the electronic band structure are exhibited in the similar manner. E_F is the Fermi energy, which is defined as that of the valence band maximum (VBM). For the *P*4*bm* phase, Bi1 and Bi3 have a considerable DOS of Bi-6*s* near the VBM while Bi2 does not. This DOS of Bi-6*s* corresponds to the antibonding states derived from the hybridization with adjacent apical O-2*p* owing to a less off-centring of Bi. Because Bi2 has a marked displacement along [001] and thereby the interaction between Bi2 and apical O atoms is weakened, Bi2-6*s* does not have a significant DOS in the valence band, as seen for Bi in the *P*4*mm* phase (**c**).

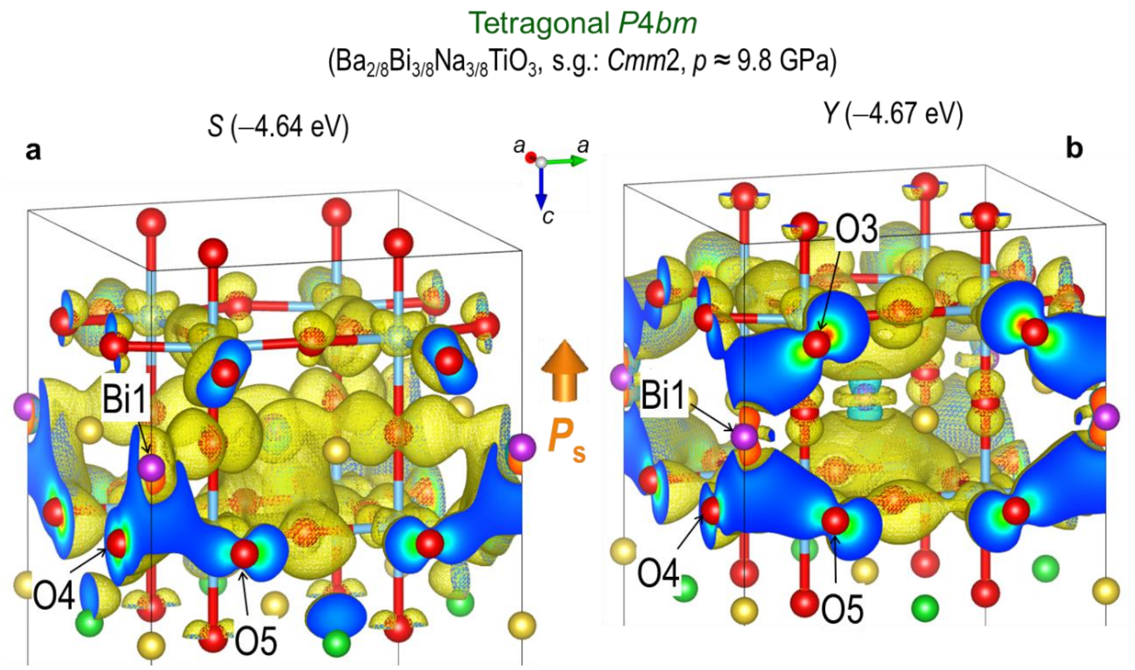

Supplementary Fig. 13 |. Wavefunction of the tetragonal *P4bm* for the $Ba_{2/8}Bi_{3/8}Na_{3/8}TiO_3$ cells. The calculations were performed for the optimized structure at $p \approx 9.8$ GPa. The wavefunctions at **a** the *S* point (–4.64 eV) and **b** the *Y* point (–4.67 eV) are shown. An increase in *p* lowers the energy of these states by 0.1–0.2 eV, which accounts for the lower free energy G_{P4bm} in the higher *p* region. These Bi1-6p_z derived occupied orbitals lead to the short bonds of Bi1-O4 and Bi1-O5, contributing to a stabilization of the *P*4*bm* phase.

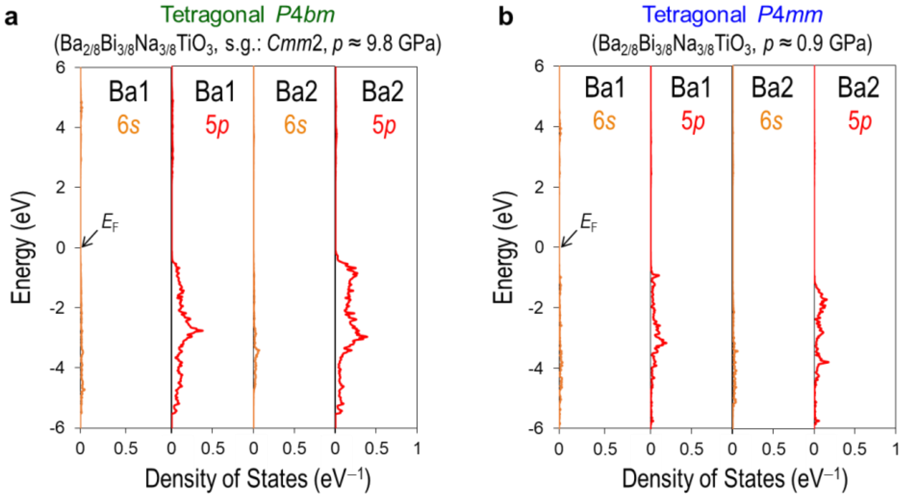

Supplementary Fig. 14 | Density of states (DOS) of Ba for the *P4bm* and *P4mm* phases. Compared with other atoms (see Supplementary Fig. 12), the partial DOS of Ba are much small in the valence band in the energy range of –6 eV to 0 eV. These results clearly show that the electronic interaction between Ba and neighbors itself does not provide a major contribution to the phase stability and also that the influence of partial Ba occupation on the A site can be regarded as an expansion of unit cell volume (V), as shown in Fig. 1. We can think that a change in V depending on the Ba composition (x) in a restricted x range, where a morphotropic phase boundary (MPB) exists, can be regarded as that on the pressure (p) for a specific model cell. In our experimental results, the two distinct MPBs; the 1st MPB and 2nd MPB are present at x ~ 5% and x ~ 7.5%, respectively. It is reasonable to consider that the 1st MPB and 2nd MPB are treated in Ba-poor and the Ba-rich structural models in DFT calculations. We, therefore, investigate the 1st MPB and 2nd MPB for the $Bi_{1/2}Na_{1/2}TiO_3$ cell and the $Ba_{2/8}Bi_{3/8}Na_{3/8}TiO_3$ cells, respectively.

Supplementary Table 1 Reported results of the structural analysis in the $(1-x)Bi_{1/2}Na_{1/2}TiO_3$–$xBaTiO_3$ system. Analytical methods are X-ray diffraction (XRD), neutron powder diffraction (NPD), Raman scattering (Raman), and transmission electron microscopy (TEM). Morphotropic phase boundary is abbreviated as MPB. While some of the papers report an irreversible phase transition after applying external stimuli, such as electric field or stress, the phases in as-prepared samples are listed.

Authors	Composition x (%)	Crystal System (Space Group)	Method
Kitanaka *et al.*, (this study)	$0 \le x \le 4$	Rhombohedral (*R*3*c*) *1	NPD
	$6 \le x \le 7$	Tetragonal (*P*4*bm*)	
	$8 \le x$	Tetragonal (*P*4*mm*)	
Takenaka *et al.*, ref. [7]	$0 \le x < 6$	Rhombohedral	XRD
	$7 \le x$	Tetragonal	
Jo *et al.*, ref. [8]	$0 \le x < 5$	Rhombohedral (*R*3*c*)	XRD
	$5 \le x < 11$	Rhombohedral (*R*3*m*)	
	$12 \le x$	Tetragonal (*P*4*mm*)	
Jo *et al.*, ref. [9]	$x = 6$	Cubic (*R*3*c* + *P*4*bm*) *2	TEM, XRD
Ma *et al.*, ref. [10]	$0 \le x \le 5$	Rhombohedral (*R*3*c*)	TEM
	$0 < x \le 6$	Rhombohedral (*R*3*c*) + Tetragonal (*P*4*bm*)	
	$6 < x < 10$	Tetragonal (*P*4*bm*)	
	$10 \le x < 11$	Tetragonal (*P*4*bm*) + Tetragonal (*P*4*mm*)	
	$12 \le x$	Tetragonal (*P*4*mm*)	
Usher *et al.*, ref. [11]	$x = 4$	Monoclinic (*Cc*) + Cubic ($Pm\bar{3}m$) *3	XRD, NPD
Garg *et al.*, ref. [12]	$5 \le x < 10$	Pseudocubic*4	XRD, NPD
Ma *et al.*, ref. [13]	$0 \le x < 3$	Monoclinic (*Cc*)	TEM
	$4 < x \le 5$	Rhombohedral (*R*3*c*)	
Garg *et al.*, ref. [14]	$0 \le x < 5$	Rhombohedral (*R*3*c*) + Monoclinic (*Cc*)	Raman, XRD, NPD
	$6 \le x < 6.75$	Cubic like	
	$7 \le x \le 10$	MPB like	
Maurya *et al.*, ref. [15]	$x = 7$	Rhombohedral (*R*3*c*) + Monoclinic*5	TEM, NPD
Ge *et al.*, ref. [16]	$0 \le x < 5$	Monoclinic (*Cc*)	XRD
	$5 \le x \le 7$	Monoclinic (*Cc*) + Tetragonal (*P*4*bm*)	
Rao *et al.*, ref. [17]	$0 \le x < 3$	Rhombohedral (*R*3*c*) + Monoclinic (*Cc*)	XRD, NPD
	$3 \le x \le 5$	Rhombohedral (*R*3*c*) + Cubic-like	
Schader *et al.*, ref. [18]	$x = 6$	Cubic ($Pm\bar{3}m$)	XRD
Mahajan *et al.*, ref. [19]	$x = 6$	Rhombohedral (*R*3*c*) + Tetragonal (*P*4*bm*)	XRD, Raman
Neagu *et al.*, ref. [20]	$x = 5$	Rhombohedral (*R*3*c*) + Tetragonal (*P*4*bm*)	TEM

*1 Our analysis does not provide a better fitting in the structural model with monoclinic *Cc*. We regard the room-temperature phase with $x \le 4$ % as rhombohedra *R*3*c* for simplicity to investigate the MPB. *2 Cubic symmetry with rhombohedral *R*3*c* and tetragonal *P*4*bm* polar nanoregions. *3 Two phase mixture of a monoclinic *Cc* phase and a minor fraction of a metrically cubic $Pm\bar{3}m$ phase. *4 Pseudocubic with long ranged modulated complex octahedral tilt. *5 Rhombohedral (*R*3*c*) with local monoclinic phase.

Supplementary Table 2 Crystallographic data of $(1–x)Bi_{1/2}Na_{1/2}TiO_3–xBaTiO_3$ at 295 K determined by the Rietveld refinements of the NPD data (Supplementary Fig. 2). See Fig. 1 for the definitions of cell deformation, rotation angle (ω), and off-center displacement (Δz).

Ba composition	x = 4 %	x = 6 %	x = 9 %
Space group	*R*3*c*	*P*4*bm*	*P*4*mm*
a / nm	0.54965 (1)	0.55197 (1)	0.38925 (1)
c / nm	1.35695 (1)	0.39035 (1)	0.39580 (1)
Cell deformation	σ = 1.0079 (1) *1	c/a_{pc} = 1.0001 (1) *2	c/a = 1.0168 (1) *2
ω / deg.	8.12 (2)	3.32 (1)	0
Δz (A-site)	0.024 (1)	0.017 (2)	0.082 (2)
Δz (B-site)	0.008 (1)	–0.022 (2)	0.046 (2)

*1 The cell deformation of the rhombohedral *R*3*c* (σ) is defined as $\sigma = \sqrt{(1+2\cos\alpha_{pc})/(1-\cos\alpha_{pc})}$, where α_{pc} is the rhombohedral angle.

*2 Tetragonalities expressed by c/a are adopted, where a_{pc} is the pseudocubic parameter, $a_{pc} = a/\sqrt{2}$, for the *P*4*bm* phase.

Supplementary Table 3 Fractional coordinates and equivalent anisotropic displacement parameters (the unit is 10^{-2} nm^2) of (1–*x*)$Bi_{1/2}Na_{1/2}TiO_3$–*x*$BaTiO_3$ at 295 K determined by the Rietveld refinements (see Supplementary Fig. 2) for **a** *x* = 4 %, **b** *x* = 6 %, and **c** *x* = 9 %.

a

atom	*x*	*y*	*z*	U^{11}	U^{22}	U^{33}	U^{12}	U^{13}	U^{23}
$Na_{0.48}Bi_{0.48}Ba_{0.04}$	0	0	0.266(1)	0.025(1)	0.025(1)	0.030(1)	0.013(1)	0	0
Ti	0	0	0	0.010(1)	0.010(1)	0.008(1)	0	0	0
O	0.126(1)	0.791(1)	0.075(1)	0.017(1)	0.019(1)	0.024(1)	0.012(1)	–0.006(1)	0.004(1)

b

atom	*x*	*y*	*z*	U^{11}	U^{22}	U^{33}	U^{12}	U^{13}	U^{23}
$Na_{0.47}Bi_{0.47}Ba_{0.06}$	0	1/2	0.537(2)	0.054(2)	0.054(2)	0.064(3)	–0.000(1)	0	0
Ti	0	0	0	0.014(1)	0.014(1)	0.008(2)	0	0	0
O_c	0	0	0.520(2)	0.038(1)	0.038(1)	0.017(2)	0	0	0
O_a	0.265(1)	0.235(1)	0.023(2)	0.029(1)	0.029(1)	0.059(2)	–0.020(1)	– 0.001(3)	0.001(3)

c

atom	*x*	*y*	*z*	U^{11}	U^{22}	U^{33}	U^{12}	U^{13}	U^{23}
$Bi_{0.455}Na_{0.455}Ba_{0.09}$	0	0	0	0.038(1)	0.038(1)	0.055(1)	0	0	0
Ti	1/2	1/2	0.464(1)	0.009(1)	0.009(1)	0.017(1)	0	0	0
O_c	1/2	1/2	−0.077(1)	0.038(1)	0.038(1)	0.012(1)	0	0	0
O_a	0	1/2	0.415(1)	0.006(1)	0.035(1)	0.046(1)	0	0	0

Supplementary Table 4 Born effective charges (Z^*) obtained by the DFPT calculations of the $Bi_{1/2}Na_{1/2}TiO_3$ and $BaTiO_3$ cells, and the weight-averaged Z_{eff}^* values of each site for their respective $Bi_{1/2}Na_{1/2}TiO_3$–$BaTiO_3$ solid solutions.

Sample*1	$Bi_{1/2}Na_{1/2}TiO_3$ cell			$BaTiO_3$ cell			Weight-averaged		
	A-site	B-site	O-site	A-site	B-site	O-site	A-site	B-site	O-site
x = 4 % (s.g.: $R3c$)	3.93 (Bi)<br>1.15 (Na)	5.22	−2.59	2.70	6.93	−3.21	2.55	5.29	−2.61
x = 6 % (s.g.: $P4bm$)	4.12 (Bi)<br>1.14 (Na)	6.10	−5.24 (O_c)<br>−1.74 (O_a)	2.84	6.93	−5.51 (O_c)<br>−2.13 (O_a)	2.64	6.16	−5.26 (O_c)<br>−1.76 (O_a)
x = 9 % (s.g.: $P4mm$)	3.89 (Bi)<br>1.15 (Na)	5.74	−4.89 (O_c)<br>−1.68 (O_a)	2.83	6.07	−4.87 (O_c)<br>−2.02 (O_a)	2.56	5.78	−4.89 (O_c)<br>−1.72 (O_a)

*1 Space group is abbreviated as "s.g."

Supplementary Table 5 Comparison of spontaneous polarization for the *R*3*c*, *P*4*bm*, and *P*4*mm* phases determined from the structural analysis in this study, along with the experimental polarization properties measured on single crystals[1,3,4].

Crystal phase	*R*3*c*	*P*4*bm*	*P*4*mm*
Spontaneous polarization [μC/cm^2]*1	38.5 (x = 4 %, //[111])	4.3 (x = 7 %, //[001])	48.8 (x = 9 %, //[001])
Remanent polarization [μC/cm^2]*2	55 (x = 0 %, //[111])	15 (x = 7 %, //[001])	54 (x = 12 %, //[001])
Coercive field [kV/cm]*2	46 (x = 0 %, //[111])	7 (x = 7 %, //[001])	30 (x = 12 %, //[001])

*1 determined by the structrual analysis.
*2 measured on single crystals.

References in Supplementary Information

1. Suzuki, M., Morishita, A., Kitanaka, Y., Noguchi, Y. & Miyayama, M. Polarization and Piezoelectric Properties of High Performance Bismuth Sodium Titanate Single Crystals Grown by High-Oxygen-Pressure Flux Method. *Jpn. J. Appl. Phys.* **49,** 09MD09 (2010).
2. Kitanaka, Y., Onozuka, H., Noguchi, Y. & Miyayama, M. High-Performance Ferroelectric $Bi_{0.5}Na_{0.5}TiO_3$ Single Crystals Grown by Top-Seeded Solution Growth Method under High-Pressure Oxygen Atmosphere. *Ferroelectrics* **414,** 24–29 (2011).
3. Kitanaka, Y. *et al.* Polarization twist in perovskite ferrielectrics. *Sci. Rep.* **6,** 32216 (2016).
4. Onozuka, H., Kitanaka, Y., Noguchi, Y. & Miyayama, M. Crystal Growth and Characterization of $(Bi_{0.5}Na_{0.5})TiO_3$–$BaTiO_3$ Single Crystals Obtained by a Top-Seeded Solution Growth Method under High-Pressure Oxygen Atmosphere. *Jpn. J. Appl. Phys.* **50,** 09NE07 (2011).
5. Cohen, R. E. Origin of ferroelectricity in perovskite oxides. *Nature* **358,** 136–138 (1992).
6. Harada, J., Pedersen, T. & Barnea, Z. X-ray and neutron diffraction study of tetragonal barium titanate. *Acta Crystallogr., Sect. A* **26,** 336–344 (1970).
7. Takenaka, T., Maruyama, K. & Sakata, K. $(Bi_{1/2}Na_{1/2})TiO_3$-$BaTiO_3$ System for Lead-Free Piezoelectric Ceramics. *Jpn. J. Appl. Phys.* **30,** 2236–2239 (1991).
8. Jo, W. *et al.* Evolving morphotropic phase boundary in lead-free $(Bi_{1/2}Na_{1/2})TiO_3$–$BaTiO_3$ piezoceramics. *J. Appl. Phys.* **109,** 14110 (2011).
9. Jo, W. *et al.* On the phase identity and its thermal evolution of lead free $(Bi_{1/2}Na_{1/2})TiO_3$-6 mol% $BaTiO_3$. *J. Appl. Phys.* **110,** 74106 (2011).
10. Ma, C., Guo, H., Beckman, S. P. & Tan, X. Creation and Destruction of Morphotropic Phase Boundaries through Electrical Poling: A Case Study of Lead-Free $(Bi_{1/2}Na_{1/2})TiO_3$-$BaTiO_3$ Piezoelectrics. *Phys. Rev. Lett.* **109,** 107602 (2012).
11. Usher, T.-M., Forrester, J. S., dela Cruz, C. R. & Jones, J. L. Crystal structure of $0.96(Na_{0.5}Bi_{0.5}TiO_3)$–$0.04(BaTiO_3)$ from combined refinement of x-ray and neutron diffraction patterns. *Appl. Phys. Lett.* **101,** 152906 (2012).
12. Garg, R., Narayana Rao, B., Senyshyn, A. & Ranjan, R. Long ranged structural modulation in the pre-morphotropic phase boundary cubic-like state of the lead-free piezoelectric $Na_{1/2}Bi_{1/2}TiO_3$-$BaTiO_3$. *J. Appl. Phys.* **114,** 234102 (2013).
13. Ma, C., Guo, H. & Tan, X. A New Phase Boundary in $(Bi_{1/2}Na_{1/2})TiO_3$−$BaTiO_3$ Revealed via a Novel Method of Electron Diffraction Analysis. *Adv. Funct. Mater.* **23,** 5261–5266 (2013).
14. Garg, R., Rao, B. N., Senyshyn, A., Krishna, P. S. R. & Ranjan, R. Lead-free piezoelectric system $(Na_{0.5}Bi_{0.5})TiO_3$-$BaTiO_3$: Equilibrium structures and irreversible structural transformations driven by electric field and mechanical impact. *Phys. Rev. B* **88,** 14103 (2013).
15. Maurya, D. *et al.* Origin of high piezoelectric response in A-site disordered morphotropic phase boundary composition of lead-free piezoelectric $0.93(Na_{0.5}Bi_{0.5})TiO_3$–$0.07BaTiO_3$. *J. Appl. Phys.*

113, 114101 (2013).

16. Ge, W. *et al.* Evolution of structure in $Na_{0.5}Bi_{0.5}TiO_3$ single crystals with $BaTiO_3$. *Appl. Phys. Lett.* **105,** 162913 (2014).

17. Rao, B. N., Avdeev, M., Kennedy, B. & Ranjan, R. Phase boundary at $x = 0.03$ and its anomalous influence on the structure and properties in the lead-free piezoelectric (1-x)$Na_{1/2}Bi_{1/2}TiO_3$-(x)$BaTiO_3$. *Phys. Rev. B* **92,** 214107 (2015).

18. Schader, F. H., Wang, Z., Hinterstein, M., Daniels, J. E. & Webber, K. G. Stress-modulated relaxor-to-ferroelectric transition in lead-free $(Na_{1/2}Bi_{1/2})TiO_3$-$BaTiO_3$ ferroelectrics. *Phys. Rev. B* **93,** 134111 (2016).

19. Mahajan, A. *et al.* Effect of Phase Transitions on Thermal Depoling in Lead-Free 0.94($Bi_{0.5}Na_{0.5}TiO_3$)–0.06($BaTiO_3$) Based Piezoelectrics. *J. Phys. Chem. C* **121,** 5709–5718 (2017).

20. Neagu, A. & Tai, C.-W. Investigation of local structural phase transitions in 95$Na_{0.5}Bi_{0.5}TiO_3$-5$BaTiO_3$ piezoceramics by means of in-situ transmission electron microscopy. *J. Appl. Phys.* **123,** 244105 (2018).
